# Pharmacokinetics and drug-likeness of antidiabetic flavonoids: Molecular docking and DFT study

**DOI:** 10.1371/journal.pone.0260853

**Published:** 2021-12-10

**Authors:** Mamaru Bitew, Tegene Desalegn, Taye B. Demissie, Anteneh Belayneh, Milkyas Endale, Rajalakshmanan Eswaramoorthy

**Affiliations:** 1 Department of Applied Chemistry, School of Applied Natural Science, Adama Science and Technology University, Adama, Ethiopia; 2 Department of Chemistry, University of Botswana, Gaborone, Botswana; 3 Department of Pharmacy, College of Health Science, Debre Markos University, Debre Markos, Ethiopia; 4 Department of Biomaterials, Saveetha Dental College and Hospitals, Saveetha Institute of Medical and Technical Sciences, Saveetha University, Chennai, India; Babasaheb Bhimrao Ambedkar University (A Central University), INDIA

## Abstract

Computer aided toxicity and pharmacokinetic prediction studies attracted the attention of pharmaceutical industries as an alternative means to predict potential drug candidates. In the present study, *in-silico* pharmacokinetic properties (ADME), drug-likeness, toxicity profiles of sixteen antidiabetic flavonoids that have ideal bidentate chelating sites for metal ion coordination were examined using SwissADME, Pro Tox II, vNN and ADMETlab web tools. Density functional theory (DFT) calculations were also employed to calculate quantum chemical descriptors of the compounds. Molecular docking studies against human alpha amylase were also conducted. The results were compared with the control drugs, metformin and acarbose. The drug-likeness prediction results showed that all flavonoids, except myricetin, were found to obey Lipinski’s rule of five for their drug like molecular nature. Pharmacokinetically, chrysin, wogonin, genistein, baicalein, and apigenin showed best absorption profile with human intestinal absorption (HIA) value of ≥ 30%, compared to the other flavonoids. Baicalein, butein, ellagic acid, eriodyctiol, Fisetin and quercetin were predicted to show carcinogenicity. The flavonoid derivatives considered in this study are predicted to be suitable molecules for CYP3A probes, except eriodyctiol which interacts with P-glycoprotein (p-gp). The toxicological endpoints prediction analysis showed that the median lethal dose (LD_50_) values range from 159–3919 mg/Kg, of which baicalein and quercetin are found to be mutagenic whereas butein is found to be the only immunotoxin. Molecular docking studies showed that the significant interaction (-7.5 to -8.3 kcal/mol) of the studied molecules in the binding pocket of the α-amylase protein relative to the control metformin with the crucial amino acids Asp 197, Glu 233, Asp 197, Glu 233, Trp 59, Tyr 62, His 101, Leu 162, Arg 195, His 299 and Leu 165. Chrysin was predicted to be a ligand with high absorption and lipophilicity with 84.6% absorption compared to metformin (78.3%). Moreover, quantum chemical, ADMET, drug-likeness and molecular docking profiles predicted that chrysin is a good bidentate ligand.

## Introduction

Computational pharmacology is a fast-growing research field focusing on the development of techniques for employing software and databases to generate and analyze molecular, biological and medical data from diverse sources [1, 2]. The methods have been used since the starting of a drug discovery to screen and identify new lead compounds available in molecular libraries [3–5], enabling parallel optimization of compound efficacy, drug-like properties and thereby playing a role in improving the quality of drug candidates [6]. The design and development of drug molecules requires earlier assessment of pharmacokinetic properties, absorption, distribution, metabolism and excretion (ADME), at a stage where multiple compounds are considered, but access to the physical samples is limited [3, 7]. Due to limited pharmacokinetics and toxicity profile of new drug-like molecules, only 11% of the drugs succeed to enter clinical development stage to reach the market [4, 8]. As a result, the pharmaceutical industries remain under immense pressure to counter the high rate of attrition in drug development that triggered an increase in the interest of computer aided toxicity and pharmacokinetic profile predictions [9]. It is evident that computer aided methods save time, human and material resources in the search of new drug-like molecules in contrast to experimental techniques [10, 11]. As a result, there has been marvelous advancement in the area of computer aided drug design and computational chemistry. These methods have been used for screening of new chemical species and their chemical properties [12]. On the other hand, web-based platforms—ADMETlab, Pro Tox-II and SwissADME—have been developed to predict ADMET properties [3, 4, 13].

Diabetes mellitus (DM) is a group of metabolic conditions in which high blood sugar levels transpire over an extended period [14–17]. Modern medical treatment methods for diabetes including injection and oral hypoglycemic drugs have some toxic or side effects, economic pressures, and so on [14, 18]. Medicinal plants have different classes of phytochemicals with bioactive compounds of various therapeutic and pharmacological potentials [19, 20], of which polyphenols are among the most studied compounds [21, 22]. The presence of bioactive phytochemical compounds such as phenols and flavonoids have been used to confirm the therapeutic usefulness of a given plant [23, 24]. Their bioactivity is originated from their structural features that give them strong tendency to scavenge free radicals, reduce oxidized chemical entities, bring metal chelation, and inhibit enzymes [25].

Metal based organic compounds that are synthesized using phytochemicals as ligands are serving as metalo-therapeutics, and they are emerging strategies to treat type II diabetes mellitus. They are known to significantly improve hyperglycemia, glucose intolerance, insulin resistance, and decrease level of serum triglyceride and plasma glucose [26, 27]. Previous studies indicated that metal-flavonoid complexes have better antioxidant activity than the free ligands alone. This is due to the attainment of extra resonating site and better stability of flavonoid metal complex radical than free flavonoids possess [16, 28]. Moreover, flavonoids and their derivatives exhibit wide spectrum of therapeutical benefits, including antidiabetic [29, 30], antiviral [26], antibacterial [31, 32], anti-inflammatory [33], cardioprotective [34], anticancer [35], antiaging [36], neuroprotective/antioxidant [35, 37, 38], antiproliferative [39] and antiestrogenic/estrogenic activities [40, 41].

In the present study, *in-silico* pharmacokinetic (ADME) properties, drug-likeness, toxicity profiles and toxicological endpoints of sixteen antidiabetic flavonoids that have ideal bidentate chelating sites for metal ion coordination were examined using SwissADME, Pro Tox II, vNN and ADMETlab web tools, Molecular docking and DFT calculations.

## Methodology

### Molecular structures

**Fig 1:**
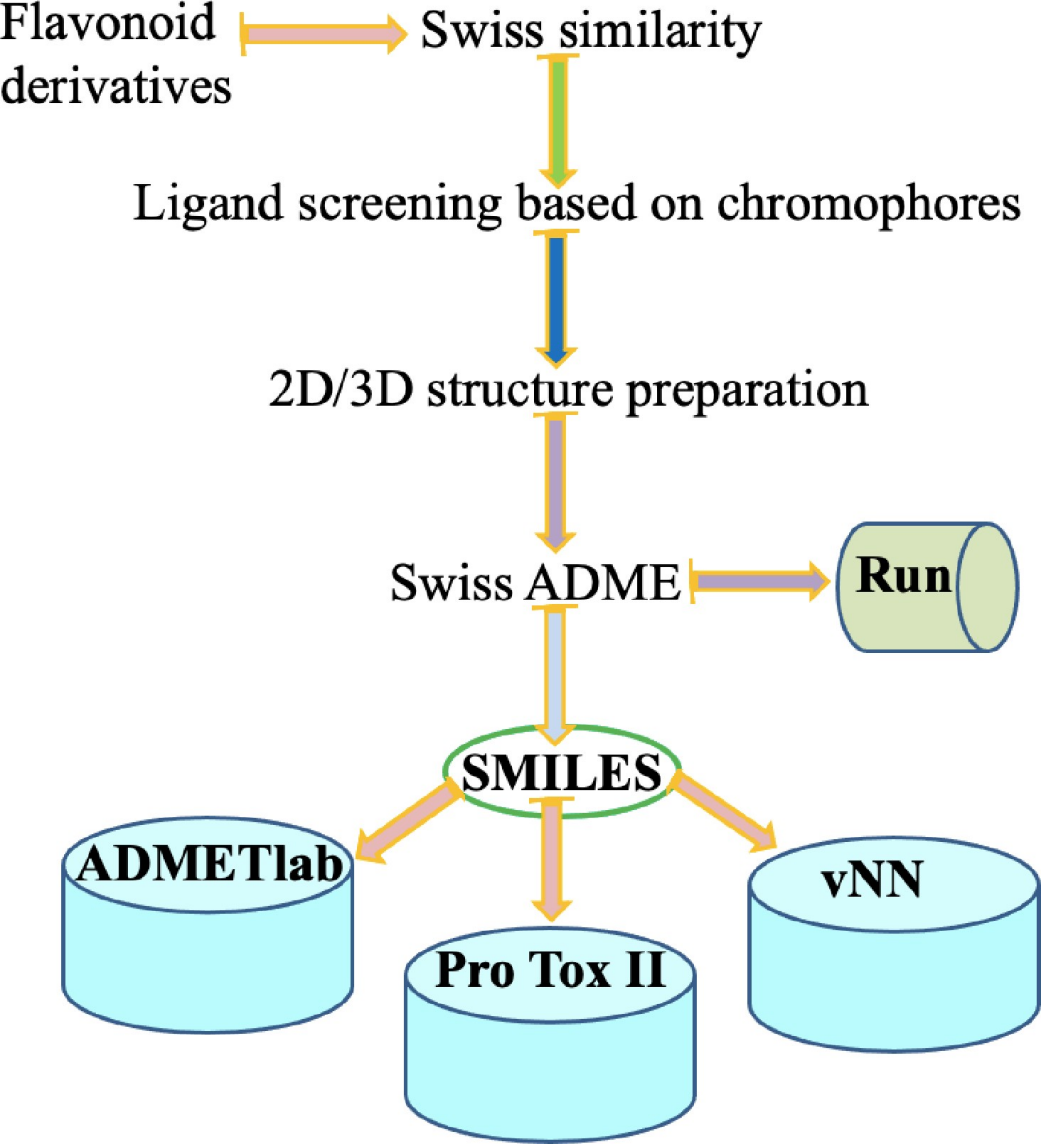
Schematic representation of ligand screening flowchart. vNN stands for variable nearest neighbor.

**Fig 2:**
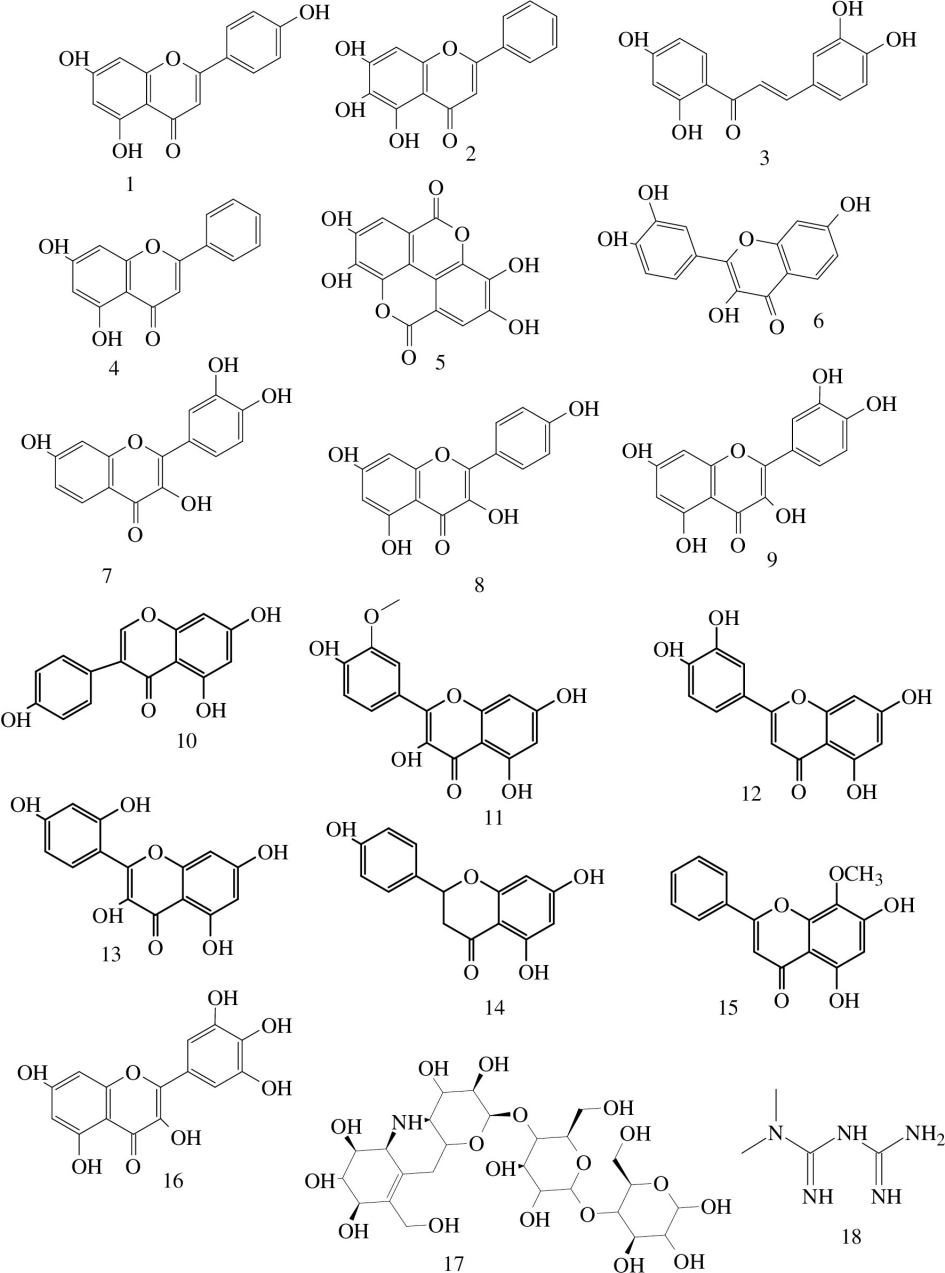
The structures of the flavonoid derivatives considered in this study. The numbers (1 to 18) stand for 1 = Apigenin, 2 = Baicalein, 3 = Butein, 4 = Chrysin, 5 = Ellagic acid, 6 = Eriodyctiol, 7 = Fisetin, 8 = Kaempferol, 9 = Quercetin, 10 = Genistein, 11 = Isorhamnetin, 12 = Luteolin, 13 = Morin, 14 = Naringenin, 15 = Wogonin, 16 = Myricetin, 17 = acarbose and 18 = metformin.

The overall procedures followed for the ligand screening process is shown in Fig 1, whereas the structures of the flavonoids studied, which are of plant origin, are presented in Fig 2. The molecules which are structurally similar with compound **9**, having a bidentate coordination cite and showed antidiabetic activity *in-vitro*, were retrieved from literature [30, 42]. The SwissADME [3] web tool from Swiss Institute of Bioinformatics (SIB) was used to convert the two dimensional structures into their simplified molecular input line entry system (SMILES) [43].

### Molecular docking studies

The molecular docking study of the target flavonoids was made by following the reported method. The 3D structure of human pancreatic α-amylase was obtained from Protein Data Bank [44] (PDB ID: 4W93). Auto Dock Vina 4.2 (MGL tools 1.5.7) with previously reported protocol was used to dock the protein Human pancreatic α-amylase and the flavonoid compounds [35–39].

### Physicochemical property, drug likeness, and pharmacokinetic predictions

Pharmaceutically significant descriptors and physically relevant properties of the target flavonoids were predicted [45, 46]. The physicochemical properties (molar refractivity, topological polar surface area, number of hydrogen bond donors/number of hydrogen bond acceptors), lipophilicity (logP_O/w_), pharmacokinetics properties (GI absorption, BBB permeation, P-gp substrate, cytochrome-P enzymes inhibition, skin permeation (log Kp)) which are critical parameters for prediction of the absorption and distribution of drugs within the body [12], and drug likeness (Lipinski’s rule of five) were predicted via SwissADME [3]. The percent absorption (% Abs) of the ligands were calculated using the reported formula % Abs = 109–0.345 TPSA [47]. The ADMETlab [4] web server was used to predict physicochemical properties (logS, logD, logP); *Absorption*: CaCO_3_ Permeability (CaCO2), P-gp inhibitor/substrate (P-gp), Human Intestinal Absorption (HIA), 20% bioavailability (F20%), and 30% bioavailability (F30%)); *Distribution*: Plasma Protein Binding (PPB), Blood Brain Barrier (BBB) and Volume Distribution (VD) and *Excretion*: Half Life (T_1/2_) and Clearance (CI). The properties of CaCO_2_, VD, PPB, CI, T_1/2_ were expressed numerically, whereas the rest of the pharmacokinetic parameters were expressed categorically [4]. Metabolism related parameter, human liver microsomal (HLM) assay was predicted using variable nearest neighbor (vNN) method [5]. Besides, water solubility, LogP and LogS values of the flavonoid derivatives were retrieved from drug bank [48], which are predicted using ALOGPS 2.1 and ChemAxon. The molecular descriptors and properties generated from all webtool/webservers were considered to screen the best candidate from the sixteen flavonoids [12].

### Toxicity prediction

A total of 19 parameters were predicted to study the toxicity profile of the sixteen flavonoids and the two controls. The toxicological endpoints (Hepatotoxicity, Carcinogenicity, Immunotoxicity, Mutagenicity) and the level of toxicity (LD_50_, mg/Kg) of the studied flavonoids were determined using ProTox-II server [13, 49]. Toxicity profiles—the hERG potassium channel inhibition (cardiotoxicity), H-HT (Human Hepatotoxicity), AMES (Ames Mutagenicity) and SkinSen (Skin sensitization)—were predicted using ADMETlab [4]. Drug induced liver injury (DILI), mitochondrial membrane potential (MMP) toxicity and cytotoxicity parameters were predicted using the vNN method [5].

### Computational details

Geometry optimizations and frequency calculations were performed using the Gaussian 09 [50] program package and the results were visualized using Gaussview 06 software. Geometries of the compounds for the density functional theory (DFT) calculations in which the DFT/B3LYP [51–53] hybrid functional was used together with 6–311++G(d, p) basis set [54]. Such an approach has been shown to give reliable results for related organic molecules [55–57]. The optimized geometries were confirmed to be real minima without any imaginary vibrational frequency by performing vibrational frequency calculations at the same level of theory. Quantum chemical descriptor set was chosen to obtain pertinent electronic and structural features [45]. The frontier molecular orbitals (FMOs), energy gap (ΔE = *E*_LUMO_-*E*_HOMO_), electronegativity (*χ* = -½ (*E*_HOMO_ + *E*_LUMO_)), electronic chemical potential (*μ* = ½ (*E*_HOMO_ + *E*_LUMO_) = -*χ*), global chemical hardness (*η* = ½ (_*E*LUMO_—*E*_HOMO_)), global softness (σ = 1/2η), global electrophilicity index (*ω* = *μ*^*2*^*/2η*), and nucleophilicity index (*Nu* = 1/*ω*), and dipole moment were also calculated and analyzed at the same level of theory [15, 58].

## Results and discussion

### Analysis of physicochemical properties and drug-likeness

Out of the sixteen flavonoids listed in Fig 2, fifteen of them do not violate Lipinski’s rule of five [59] for oral availability, demonstrating that the compounds have drug-like molecular nature (Table 1) except myricetin (**16**) holding six NHBDs (> 5). It is important to note that Lipinski’s rule of five is essential for rational drug design and it has been suggested that the low permeability or poor absorption for a given compounds results when it violates one of Lipinski’s rule of five [12]. Molecular weight, LogP and number of hydrogen bond acceptors (NHBAs) of all flavonoids are within the recognized values of less than 500, 3 and 10, respectively. It has been predicted that the molecules that have similar molecular mass (Apigenin (**1**) and Baicalein (**2**); Fisetin (**7**) and Kaempferol (**8**); Isorhamnetin (**11**), Luteolin (**12**), and Naringenin (**14**)) showed similarities in TPSA, %Abs and MR (Table 1). The molecules with a TPSA of 140 Å^2^ and above would be poorly absorbed (< 10% fractional absorption), while those with a TPSA 60 Å^2^ would be well absorbed (> 90%) [60]. The TPSA and %Abn of the flavonoid derivatives showed the preference of apigenin (**1**), baicalein (**3**), chrysin (**4**), genistein (**10**), and wogonin (**15**) with better and comparable percent absorption to the control metformin (**18**). It is found that the %Abn and TPSA values follow the order chrysin (**4**) > wogonin (**15**) > metformin (**18**) > genistein (**10**) = apigenin (**1**) = baicalein (**2**).

**Table 1:** The physicochemical property of the flavonoids and the two control drugs.

	Physicochemical Property								
Flavonoid	MW	NHBAs	NHBDs	TPSA	%Abs	MR	log S	LogD	LogP
**1**	270.24	5	3	90.9	77.64	73.99	-3.595	0.487	2.577
**2**	270.24	5	3	90.90	77.64	73.99	-3.578	0.583	2.577
**3**	272.25	5	4	97.99	75.19	74.34	-3.427	0.266	2.405
**4**	254.24	4	2	70.67	84.62	71.97	-3.709	0.847	2.871
**5**	302.19	8	4	141.34	60.24	75.31	-3.076	-0.740	1.313
**6**	288.25	6	4	107.22	72.01	73.59	-3.534	0.311	2.216
**7**	286.24	6	4	111.13	70.66	76.01	-3.417	0.284	2.282
**8**	286.24	6	4	111.13	70.66	76.01	-3.427	0.273	2.282
**9**	302.24	7	5	131.36	63.68	78.03	-3.534	0.142	1.988
**10**	270.24	5	3	90.90	77.64	73.99	-3.632	0.563	2.577
**11**	286.24	6	4	111.13	70.66	76.01	-3.427	0.273	2.282
**12**	286.24	6	4	111.13	70.66	76.01	-3.502	0.302	2.282
**13**	302.24	7	5	131.36	63.68	78.04	-3.282	0.113	1.988
**14**	286.24	6	4	111.13	70.66	76.01	-3.427	0.273	2.282
**15**	284.26	5	2	79.90	81.43	78.46	-3.696	0.927	2.88
**16**	318.24	8	6	151.59	56.70	80.06	-3.220	-0.068	1.694
17	645.6	19	14	321.17	-1.80	136.69	-1.368	2.543	-8.565
18	129.16	2	4	88.99	78.30	36.93	-0.656	-0.192	-1.034

The results also showed an inverse relationship between TPSA and %Abs, and direct relationship between %Abs and MR. The analysis of the %Abs of the sixteen studied flavonoids and the two controls for their percent absorption (Table 1) showed that chrysin (**4**) have the highest percent absorption (84.6%) than all the other flavonoids and the two controls, whereas metformin (**18**) displayed better percent absorption (78.3%) than acarbose. These results are in good agreement with a previous report on pharmacokinetic properties of apigenin (**1**), eriodyctiol (**6**), fisetin (**7**), kaempferol (**8**) and quercetin (**9**) via molinspiration and pkCSM servers [61].

The calculated result of LogS showed that chrysin (**4**) (-3.709) is the least soluble molecule and ellagic acid (**5**) (-3.076) has the highest solubility. The relatively high solubility of ellagic acid (**5**) is due to the presence of four hydroxyl and two ketone pharmacophores [46] relative to the other flavonoids studied in this work. The computed results for the distribution coefficient (logD_7.4_) of the flavonoids (Table 1) showed that they have high solubility with low permeability by passive transcellular diffusion [62]. It is predicted that acarbose (**17**) has moderate solubility, moderate permeability and low metabolism with logD_7.4_ value of 2.543 and very low lipophilicity of -8.565.

The distribution coefficients (logP) of the flavonoids (Tables 1 and 2) range between 1.313 and 2.871. The results of the logP values showed that they have optimal lipophilicity (Optimal: 0 < LogP < 3). The logP results for the two controls, acarbose (**17**) and metformin (**18**), are less than zero, -1.034 and -8.565, respectively, suggesting that the two compounds have poor membrane permeability. The logP of apigenin (**1**) (3.07) and chrysin (**4**) (3.44) were found to be greater than three as predicted in ALOGPS (Table 2) which might be due to the small number of hydroxyl groups present in both compounds.

**Table 2:** Comparison of solubility, partition coefficient, water solubility, and P^Ka^ of flavonoids predicted using different methods.

*Molecule*	*LogS*		*Water solubility*	*Partition coefficient (Log P)*			*Pka*
	Method			Method			ChemAxon
	ALOGPS	ADMETlab	Pred. (mg/mL)	ChemAxon	ALOGPS	ADMETlab	
**1**	-3.4	-3.595	0.118	2.71	3.07	2.577	6.63
**2**	-	-3.578	-	-	-	2.577	-
**3**	-	-3.427	-	-	-	2.405	-
**4**	-3.4	-3.709	0.105	3.01	3.44	2.871	6.58
**5**	-2.6	-3.076	0.823	2.32	1.59	1.313	5.54
**6**	-	-3.534	-	-	-	2.216	-
**7**	-3.3	-3.417	0.151	1.81	2.03	2.282	6.32
**8**	-3.2	-3.427	0.178	2.46	1.99	2.282	6.44
**9**	-3.1	-3.534	0.261	2.16	1.81	1.988	6.44
**10**	-3.3	-3.632	0.123	3.08	3.04	2.577	6.55
**11**		-3.427	-	-	-	2.282	-
**12**	-3.3	-3.502	0.138	2.4	2.73	2.282	6.57
**13**		-3.282	-	-	-	1.988	-
**14**	-3.1	-3.427	0.214	2.84	2.47	2.282	7.91
**15**		-3.696	-	-	-	2.880	-
**16**	-3	-3.22	0.301	1.85	1.66	1.694	6.43

The water solubility (mg/mL) of some of the flavonoids considered in this work was retrieved from the drug bank (predicted using ALOGPS) and tabulated in Table 2. It is predicted that ellagic acid (**5**) has high solubility (0.823 mg/mL), whereas chrysin (**4**) is found to have low solubility (0.105 mg/mL). These results are in agreement with those predicted using ADMETlab as well as with the experimental findings suggesting that chrysin (**4**) has low aqueous solubility and high absorption or absorption enhancer role [63].

The p*Ka* results range from 5.54 to 7.91 indicating that the studied flavonoids fall under the category of neutral organic acids, in good agreement with experimental p*Ka* of apigenin (**1**), ellagic acid (**5**) and quercetin (**9**) (7.12, 6.69, and 7.17, respectively). The relatively high p*Ka* of naringenin (**14**) (7.91), apigenin (**1**) (6.63), chrysin (**4**) (6.58), luteolin (**12**) (6.57) and genistein (**10**) (6.55) may cause difficulty of deprotonation, suggesting that the ligands have stronger coordination ability with metals [64].

On the other hand, the lipophilicity (LogP_o/w_) study showed that wogonin (**15**) (2.880), chrysin (**4**) (2.871), genistein (**10**) (2.557), apigenin (**1**) and **2** (2.577) are highly lipophilic than the other molecules, whereas quercetin (**9**) and morin (**13**) (1.988) were found to be less lipophilic ligands next to ellagic acid (**5**) (1.313) (Table 2). The presence of one methoxy group in the benzoyl part of wogonin (**15**) makes it more lipophilic than chrysin (**4**). It has also been found that the structural resemblance in benzoyl or cinnamoyl part of the flavonoid derivatives play a role in their physicochemical properties. The calculated LogP_o/w_ of the flavonoid derivatives considered in this work is found to range between 1.313 and 2.880. Our predicted results are in good agreement with the previously reported *P*_o/w_ values of 1.92, 2.27, 3.11 and 1.82 for apigenin (**1**), eriodyctiol (**6**), fisetin (**7**) and kaempferol (**8**), respectively [3]. Although further clinical study is required, the predicted results suggest that oral and intestinal absorptions are possible.

## Pharmacokinetics properties

### Absorption

To evaluate the absorption property of the flavonoids, we predicted Caco-2-Permeability (Papp), P-glycoprotein (P-gp), substrate or inhibitor (P-gpinh/P-gpsub), Human intestinal absorption (HIA), 20% bioavailability (F_20_), and 30% bioavailability (F_30_) as presented (Table 3). It is found that the values of Caco-2-Permeability are in the range from -4.732 to -6.63 cm/s, suggesting they have low to moderate permeability. The high negative Caco-2-Permeability (cm/s) of myricetin (**16**) (-6.63 cm/s), morin (**13**) (-6.206 cm/s), kaempferol (**8**) (-6.163 cm/s) and ellagic acid (**5**) (-6.001 cm/s) is mainly due to the presence of large number of NHBDs and NHBAs in their structures which have the potential to rendere their permeability. The two controls, acarbose (**17**) and metformin (**18**) are predicted to have Caco-2 permeability values of -5.803 cm/s and -7.128 cm/s, respectively. The predicted HIA data of the sixteen flavonoids and the two control drugs are represented categorically as 0 and 1 (Table 3) which showed that apigenin (**1**), chrysin (**4**), fisetin (**7**), genistein (**10**), wogonin (**15**), and metformin (**18**) have high HIA (≥ 30%) whereas acarbose (**17**) and the rest six flavonoids are predicted to have HIA below 30% (Table 3). Flavonoids baicalein **(2)**, chrysin **(4)**, genistein **(10)**, wogonin **(15)** and the control, metformin (**18)** predicted to show bioavailability greater than 30%, whereas the rest of the flavonoids are predicted to show bioavailability between 20 to 30%. Acarbose (**17**) is known for its extremely low bioavailability and less than 2% of absorption as active drug [48]. We predicted low bioavailability (<20%, Table 3) and extremely low percent absorption of acarbose (-1.8%, Table 1).

**Table 3:** Absorption, distribution and excretion parameters of the flavonoids and the two controls.

Flavonoids	Absorption						Distribution			Excretion	
	Caco-2	Pgp inh	Pgp sub	HIA	F20	F30	PPB	BBB	VD	T1/2	CL
**1**	-4.985	0	0	1	1	0	90.03	0	-0.58	1.358	1.90
**2**	-5.120	1	0	0	1	1	85.90	1	-0.69	1.259	1.92
**3**	-5.204	0	0	0	1	0	86.74	0	-0.92	1.102	1.66
**4**	-4.973	0	0	1	1	1	89.57	1	-0.35	1.667	1.98
**5**	-6.001	0	0	0	0	0	65.67	1	-0.99	1.109	1.43
**6**	-5.302	0	0	0	1	0	92.20	1	-0.91	0.634	1.89
**7**	-5.126	0	0	1	1	0	91.06	0	-0.95	0.586	1.90
**8**	-5.075	0	0	0	1	0	90.85	0	-0.98	0.696	1.90
**9**	-6.168	0	0	0	1	0	94.99	0	-1.56	0.200	2.00
**10**	-4.999	0	0	1	1	1	90.537	0	-0.58	1.299	1.941
**11**	-5.075	0	0	0	1	0	90.847	0	-0.97	0.696	1.941
**12**	-5.123	0	0	0	1	0	91.796	0	-0.93	0.745	1.919
**13**	-6.206	0	0	0	1	0	87.95	0	-1.46	0.714	2.002
**14**	-5.075	0	0	0	1	0	90.847	0	-0.97	0.696	1.941
**15**	-4.732	1	0	1	1	1	86.612	1	-0.32	1.706	1.894
**16**	-6.630	0	0	0	1	0	76.595	0	-1.39	0.940	1.709
17	-7.128	0	0	0	0	0	21.14	0	-1.22	1.320	0.50
18	-5.803	0	0	1	1	1	23.65	1	-0.19	1.806	0.90

### Distribution

Plasma Protein Binding (PPB), Blood Brain Barrier (BBB) permeability, and Volume of distribution (VD) were parameters considered to study the distribution of drug candidates (Table 4). The PPB of apigenin (**1**), eriodyctiol (**5**), fisetin (**7**), kaempferol (**8**) and quercetin (**9**) are predicted to be above 90% to bind to common blood proteins significantly, indicating that the pharmacologic effect of these compounds is low. The control drugs, acarbose (**17**) and metformin (**18**) weakly bind to common blood proteins. This could be one of the reasons that make acarbose as a working drug, while it has very low percent absorption (-1.8%).

**Table 4:** The interaction of target flavonoids with cytochromes P450 isoforms predicted using SwissADME.

Molecule	GI abn	BBB prn	p-gp substrate	CYP 1A2 In	CYP 2C19 In	CYP 2C9 In	CYP 2D6 In	CYP 3A4 In	log Kp (cm/s)	HLM	MMP
**1**	High	No	No	Yes	No	No	No	Yes	-5.8	√	√
**2**	High	No	No	Yes	No	No	Yes	Yes	-5.7	Ø	√
**3**	High	No	No	Yes	No	Yes	No	Yes	-5.96	Ø	√
**4**	High	Yes	No	Yes	No	No	Yes	Yes	-5.35	Ø	√
**5**	High	No	No	Yes	No	No	No	No	-7.36	Ø	X
**6**	High	No	Yes	No	No	No	No	Yes	-6.62	Ø	Ø
**7**	High	No	No	Yes	No	No	Yes	Yes	-6.65	Ø	√
**8**	High	No	No	Yes	No	No	Yes	Yes	-6.7	Ø	√
**9**	High	No	No	Yes	No	No	Yes	Yes	-7.05	Ø	√
**10**	High	No	No	Yes	No	No	Yes	Yes	-6.05	Ø	√
**11**	High	No	No	Yes	No	No	Yes	Yes	-6.7	Ø	√
**12**	High	No	No	Yes	No	No	Yes	Yes	-6.25	Ø	√
**13**	High	No	No	Yes	No	No	Yes	Yes	-7.05	Ø	√
**14**	High	No	No	Yes	No	No	Yes	Yes	-6.7	Ø	√
**15**	High	No	No	Yes	No	Yes	Yes	Yes	-5.56	Ø	√
**16**	Low	No	No	Yes	No	No	No	Yes	-7.4	Ø	√
**17**	Low	No	Yes	No	No	No	No	No	-16.29	Ø	X
**18**	High	No	No	No	No	No	No	No	-7.84	Ø	Ø

logKp skin permeation, abn = absorption, prn = permission, In = Inhibitor, GI = gastrointestinal, BBB = blood brain barrier, In = inhibitor. Symbols X, √ and Ø represents inactive, active and no high confidence prediction available, respectively.

Baicalein (**2**), chrysin (**4**), ellagic acid (**5**), eriodyctiol (**6**), wogonin (**15**) and metformin (**18**) were predicted to permeate BBB using ADMETlab. However, our prediction using SwissADME webtool showed that only chrysin (**4**) can permeate BBB. On contrary to previous report on ellagic acid (**5**) studied using pkCSM server [6], our study showed positive result predicted by ADMETlab for ellagic acid (**5**) to permeate BBB.

The volume of distribution (VD) in L/Kg were predicted to be all negative, indicating higher distribution of the flavonoid derivatives and the two control drugs in plasma than in tissues, confined to plasma proteins. The hydrophilic character of the flavonoids contributed to low values of VD. The more negative value of VD for quercetin (-1.56) and less negative value for wogonin (-0.32) followed by chrysin (-0.35) among flavonoids were also presented in Table 3. The high VD values of wogonin (**15**) and chrysin (**4**)—relative to the other flavonoids -showed the consistency of absorption and distribution parameters.

### Metabolism and excretion

The skin permeability, Kp, values obtained for all the flavonoid derivatives considered in this study are in the range of -7.4 to -5.35 cm/s (Table 4) inferring low skin permeability [3]. It is found that log Kp increases (more negative) with the increase in molecular size (Table 4). The results indicated that eriodyctiol (**6**) is the only flavonoid to act as drug transporter of P-glycoprotein substrate from target flavonoids considered (Table 4). This may lead to pharmacokinetics-related drug-drug interactions leading to toxic or other unwanted adverse effects due to the lower clearance and accumulation of the drug or its metabolites [65]. In contrary, it is the only molecule that can act as CYP1A2 inhibitor (Table 4). Its interaction with p-gp together with its carcinogenicity will lead eriodyctiol (**6**) to be excluded from the list of bidentate ligands for metal complexation. Except ellagic acid (**5**), the rest of the flavonoid derivatives considered in this study are predicted to interact with CYP3A, making them very suitable molecules as CYP3A4 probes (Table 4). CYP3A enzymes are important determinants in the therapeutic efficacy and toxicity of numerous drugs. The interactions at the CYP3A level are often the cause of pronounced drug-drug interactions [66]. Baicalein (**2**), chrysin (**4**), fisetin (**7**), kaempferol (**8**), quercetin (**9**), genistein (**10**), isorhamnetin (**11**), luteolin (**12**), morin (**15**), naringenin (**14**) and wogonin (**15**) are predicted as D6 inhibitors. Previous study by May & Schindler, [67] showed that the interaction of antidiabetic drugs with other drugs in CYP3A4 and CYP2D6 inhibition mechanism has an additive effect for antidiabetic drugs [67]. In the present work, all the flavonoid derivatives except ellagic acid (**5**) were found to inhibit CYP3A4. These results suggest that the flavonoids studied in this work potentially have antidiabetic effect in agreement with previous reports [42, 68]. In this study, only butein (**3**) and wogonin (**15**) are predicted to interact with 2C9. Previous report indicated that the inhibition and induction of the CYP enzymes are probably the common causes for most drug interactions [65]. Hence, the predicted carcinogenicity and immunotoxicity (Table 5) together with its interaction with 2C9 would lead butein as a flavonoid derivative with high safety risk. In general, our predicted results could be used as important inputs for further experimental studies.

**Table 5:** Pro Tox II predicted organ toxicity, toxicological end points and acute toxicity.

Flavonoid	Hepatotoxicity	Carcinogenicity	Immunotoxicity	Mutagenicity	Cytotoxicity	LD_50_(mg/Kg)	Acute Toxicity class
**1**	inactive	inactive	inactive	inactive	inactive	2000	4
**2**	inactive	Active	inactive	active	inactive	3919	5
**3**	inactive	Active	active	inactive	inactive	1000	4
**4**	inactive	inactive	inactive	inactive	inactive	2500	5
**5**	inactive	Active	inactive	inactive	inactive	2991	4
**6**	inactive	Active	inactive	inactive	inactive	2000	4
**7**	inactive	Active	inactive	inactive	inactive	159	3
**8**	inactive	inactive	inactive	inactive	inactive	3919	5
**9**	inactive	Active	inactive	active	inactive	159	3
**10**	inactive	inactive	inactive	inactive	inactive	2500	5
**11**	inactive	inactive	inactive	inactive	inactive	3919	5
**12**	inactive	Active	inactive	active	inactive	3919	5
**13**	inactive	inactive	active	inactive	inactive	3919	5
**14**	inactive	inactive	inactive	inactive	inactive	3919	5
**15**	inactive	inactive	inactive	inactive	inactive	3919	5
**16**	inactive	Active	inactive	active	inactive	159	3
**18**	Inactive	Inactive	inactive	inactive	inactive	1300	4

The human liver microsomal (HLM) stability and mitochondrial membrane potential (MMP) parameters of the flavonoid derivatives predicted via the vNN method (Table 4) showed that most of the flavonoid derivatives showed mitochondrial toxicity. Ellagic acid (**5**) and acarbose (**17**) were predicted to be inactive for MMP. Apigenin (**1**) was active for HLM prediction, and could be metabolized rapidly by the liver that will reduce its effective therapeutic concentration in the body [5].

### Toxicity profile/toxicological endpoints

Absence of toxicity is one of the major factors for selecting a compound as a therapeutic candidate [69]. Herein, the organ toxicity (hepatotoxicity) and toxicological endpoints (carcinogenicity, immunotoxicity, mutagenicity and cytotoxicity) of the sixteen flavonoid derivatives were predicted. The predicted descriptors are presented in Table 5. The results show that all of the compounds are not hepatotoxic and cytotoxic. Of the considered flavonoid derivatives, baicalein (**2**), myricetin (**16**), luteolin (**12**) and quercetin (**9**) and butein (**3**) show two toxicological endpoints; carcinogenicity and mutagenicity, and carcinogenicity and immunotoxicity, respectively. On the other hand, ellagic acid (**5**), eriodyctiol (**6**), fisetin (7) and morin (**15**) show one toxicological endpoint (carcinogenicity). Apigenin (**1**), chrysin (**4**), kaempferol (**8**), wogonin (**15**), naringenin (**14**), isorhamnetin (**11**), and genistein (**10**) are free from any of the predicted toxicological endpoints. Acute toxicity predictions indicate that the flavonoids considered are not fetal; however, they can be categorized as harmful toxic classes.

The median lethal dose (LD_50_) values were found to be in the range from 159–3919 mg/Kg (Table 5). According to the globally harmonized system of classification of labeling of chemicals (as described in Pro Tox II), quercetin (**9**), fisetin (**7**), and myricetin (**16**) are predicted as toxic (Class III), whereas apigenin (**1**), butein (**3**), elagic acid (**5**) and eriodyctiol (**6**) are harmful (Class IV) and the rest were categorized as “may be harmful” (Class V) toxicity classes. The results show that most of the drug-like compounds have the tendency to have carcinogenic activities than any toxicological endpoints.

Besides the ADMETlab descriptors, vNN descriptors such as hERG Blockers (hERG), human hepatotoxicity (H-HT), Ames Mutagenicity (AMES), Skin sensitization (SkinSen) were used to predict the toxicity of the flavonoid derivatives and the two controls. The results are presented in Table 6. The numbers 1 and 0 in the category section (Table 6) stand for positive and negative, respectively, for the predicted toxicities. Except apigenin (**1**), baicalein (**2**) and genistein (**10**), the rest of the flavonoid derivatives and the two control drugs were not predicted to be human ether-a-go-go related gene (hERG) blockers. However, most of them were predicted to show H-HT and mutagenicity (Table 6).

**Table 6:** Toxicity profile of flavonoids and the two controls predicted using different methods.

	Toxicity					
Flavonoids considered	ADMETlab					vNN
	hERG	H-HT	AMES	SkinSen	DILI	Cytotoxicity
**1**	1	1	0	0	X	X
**2**	1	1	1	0	Ø	X
**3**	0	1	0	0	Ø	Ø
**4**	0	0	0	0	Ø	X
**5**	0	1	0	0	Ø	Ø
**6**	0	0	1	0	Ø	Ø
**7**	0	1	1	0	Ø	X
**8**	0	1	1	0	Ø	X
**9**	0	1	1	0	Ø	X
**10**	1	1	0	0	Ø	Ø
**11**	0	1	1	0	Ø	X
**12**	0	1	1	0	Ø	X
**13**	0	0	1	0	Ø	X
**14**	0	1	1	0	Ø	X
**15**	0	1	0	0	Ø	X
**16**	0	0	1	0	Ø	X
**17**	0	0	0	0	√	Ø
**18**	0	1	0	1	Ø	Ø

The symbols X, √ and Ø represents inactive, active and no high confidence prediction available, respectively.

The vNN predicted drug induced liver injury (DILI) and cytotoxicity results (Table 6) showed that most of the flavonoid derivatives are not cytotoxic which is in line with results from Pro Tox II prediction. Apigenin (**1**) and acarbose (**17**) were predicted with inactive and active, respectively, to induce liver injury. The results also indicate the tendency of acarbose (**17**) to cause DILI (Table 6), which is in agreement with previous report on the risk of hepatotoxicity of acarbose (**17**) [70] and the probability of acarbose-induced hepatotoxicity [71].

### Density functional theory analysis

The frontier molecular orbital analysis (HOMO-LUMO) was performed to predict the reactivity of the flavonoid derivatives. DFT chemical descriptors: electronegativity (*χ*), electronic chemical potential (*μ)*, globalchemical hardness (*η)*, global softness (σ), global electrophilicity index (*ω)* and nucleophilicity index *(Nu)* were derived from the energy gap (Eg = ELUMO—E_HOMO_), are presented in Table 7. Density functional theory-based quantum chemical descriptors (DFTB-QCDs) help to identify the associations between chemical structures and properties, and play an essential role in chemistry, environment, pharmacology, and health science research [72, 73]. The quantum chemical descriptors such as E_HOMO_ and E_LUMO_ play an important role in predicting the energy gap of molecules and determining the anti-oxidant, reactivity, hardness and softness of molecules [73]. The calculated energy gap between HOMO and LUMU for the studied flavonoid derivatives were in the range from 2.428 eV (chrysin (**4**)) to 7.982eV (genistein (**10**)), whereas metformin (**18**) has the largest band gap. The high HOMO energy (-4.734 eV), the small energy gap (2.428 eV), small chemical hardness (0.045 eV), and large softness (11.207 eV) calculated for chrysin (**4**) molecule makes the molecule a good candidate as a chelating agent. On the other hand, the high dipole moment (8.293 Debyee) value found for chrysin (**4**) makes the molecule a preferred ligand for biological activity (antimicrobial). Among the 16 flavonoid derivatives, the highest HOMO-LUMO energy gap, hardness, and high nucleophilicity were predicted for genistein (**10**) (Table 7).

**Table 7:** Quantum chemical descriptors of the flavonoid derivatives calculated using DFT/B3LYP hybrid functional was used together with 6–311++G(d,p) basis set.

FLs	*Eg (eV)*	*χ*	*μ*	*Ƞ*	*Σ*	*ω*	*Nu*	*Dipole Moment*
**1**	4.190	0.155	-0.155	0.077	6.494	0.156	6.393	6.679
**2**	4.114	0.158	-0.158	0.076	6.615	0.165	6.043	7.272
**3**	2.838	0.180	-0.180	0.052	9.587	0.309	3.234	4.336
**4**	2.428	0.129	-0.129	0.045	11.207	0.187	5.333	8.293
**5**	4.339	0.172	-0.172	0.080	6.272	0.185	5.413	1.976
**6**	4.142	0.156	-0.156	0.076	6.569	0.160	6.232	5.520
**7**	3.764	0.157	-0.157	0.069	7.230	0.178	5.616	5.143
**8**	3.867	0.154	-0.154	0.071	7.036	0.166	6.011	6.342
**9**	3.794	0.157	-0.157	0.070	7.173	0.178	5.627	6.225
**10**	7.982	0.063	-0.063	0.147	3.409	0.013	74.683	8.927
**11**	3.867	0.154	-0.154	0.071	7.037	0.166	6.013	6.339
**12**	4.082	0.157	-0.157	0.075	6.666	0.165	6.065	7.019
**13**	3.787	0.159	-0.159	0.070	7.185	0.181	5.539	4.287
**14**	3.869	0.154	-0.154	0.071	7.034	0.166	6.024	6.269
**15**	3.956	0.159	-0.159	0.073	6.879	0.173	5.765	8.622
**16**	3.812	0.156	-0.156	0.070	7.138	0.174	5.754	6.364
**18**	14.281	0.047	-0.047	0.262	1.905	0.004	237.574	5.795

Based on the quantum chemical reactivity descriptors, the isoflavone class of flavonoids, genistein (**10**) has the highest chemical hardness (0.147 eV) and the lowest electrophilicity (0.013 eV). Whereas the highest electrophilicity (0.309 eV) and the lowest chemical hardness (0.052 eV), next to chrysin (**4**) (0.045 eV), was predicted for the chalcone family, butein (**3**). The electrophilicity, electronegativity and softness quantum chemical descriptors for apigenin (**1**)/genistein (**10**) were found to be 0.077/0.147 eV, 6.494/3.409 eV and 0.156/0.013 eV, respectively, indicating good chelating nature of apigenin (**1**) over genistein (**10**).

### Molecular docking evaluation

**Fig 3:**
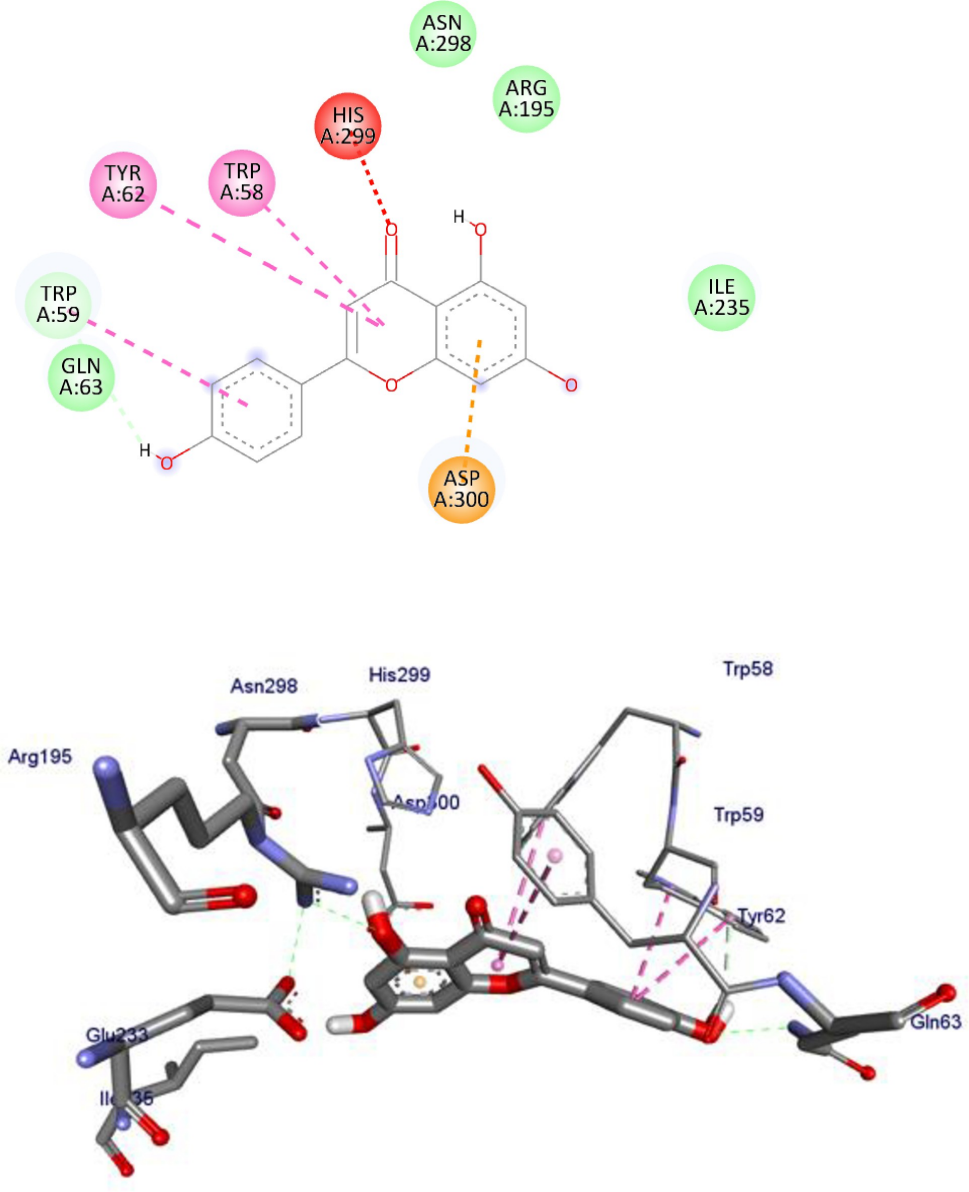
2D (upper panel) and 3D (upper panel) representations of the binding modes of compounds apigenin to Human α-amylase (PDB ID: 4W93).

**Fig 4:**
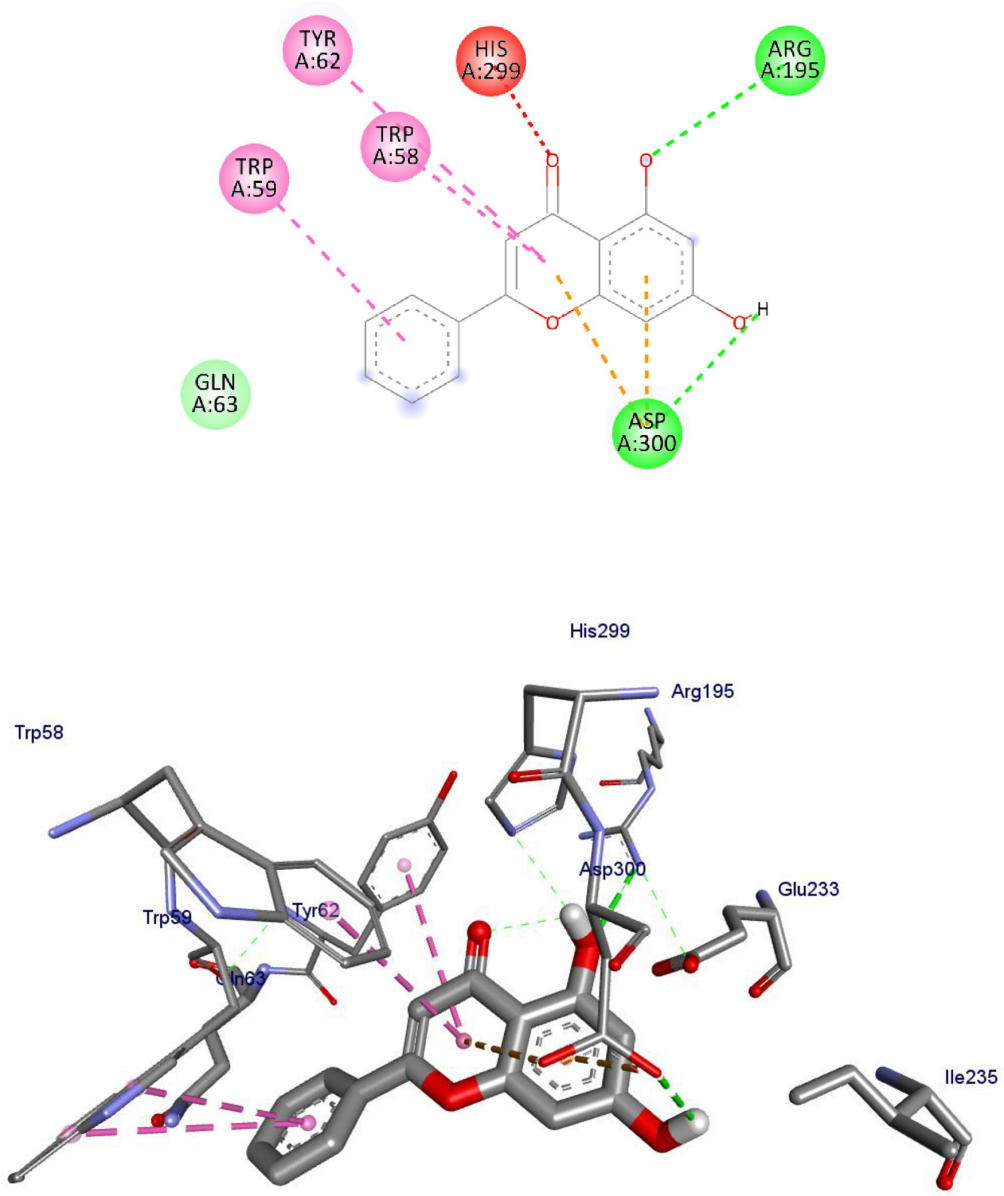
2D (upper panel) and 3D (lower panel) representations of the binding modes of chrysin to Human α-amylase (PDB ID: 4W93).

**Fig 5:**
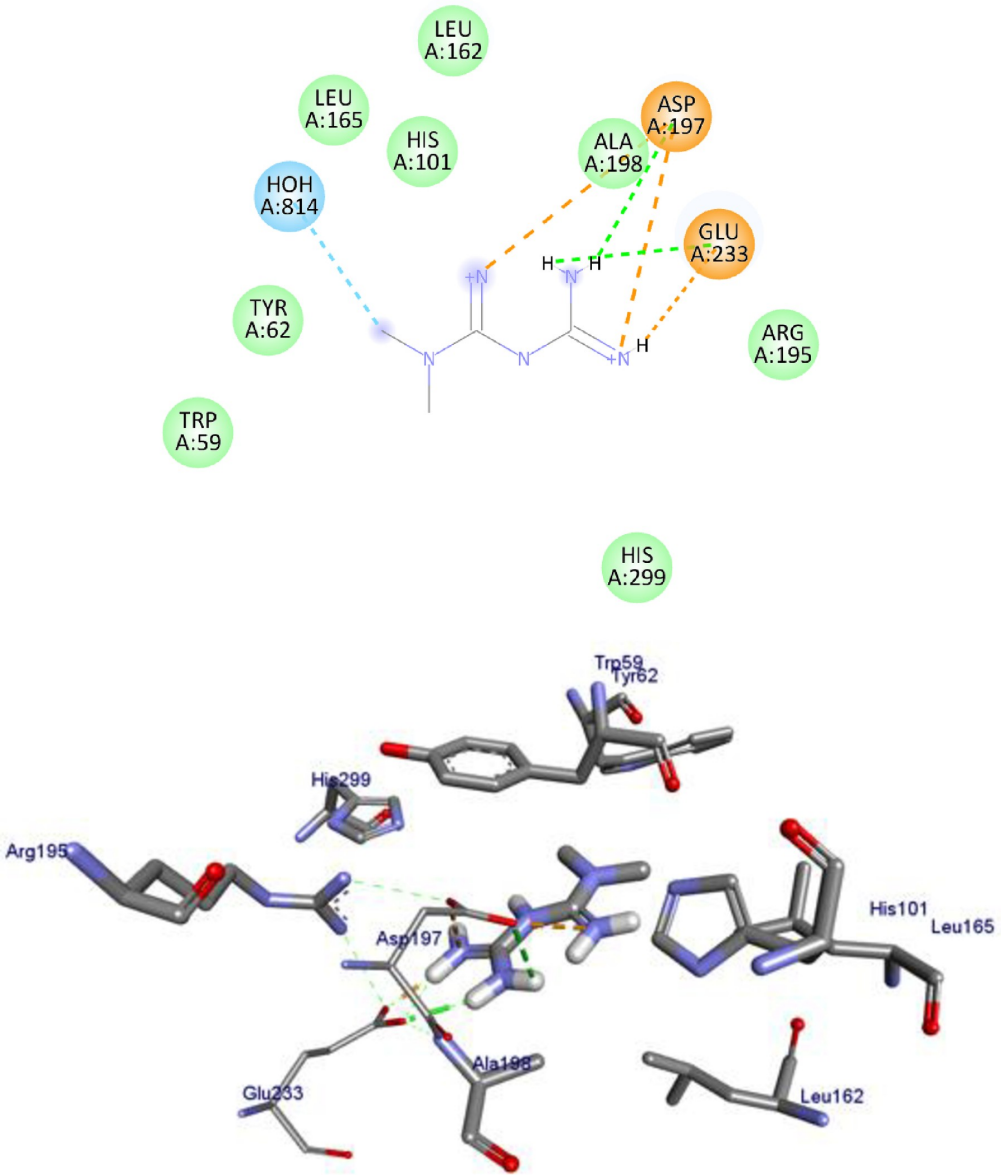
2D (upper panel) and 3D (lower panel) representations of the binding modes of metformin to Human α-amylase (PDB ID: 4W93).

α-Amylase has been a pivotal target in anti-diabetic drug development. In the present work, we studied the interactions between the Human α-amylase (PDB ID: 4w93) protein against the flavonoids and compared the results against the clinical drug metformin. The 2D and 3D representations of apigenin (**1**)-, chrysin (**4**)- and the control metformin (**18**)-protein interactions are given from Figs 3–5, and the rest of flavonoids are given in S1-S7 Figs in S1 File. The binding affinity, H-bond and residual interaction of flavonoids and metformin (**18**) (anti-diabetic drug) were summarized in Table 8. Compared to metformin (**18**), the flavonoid compounds shown significant interaction within the active site of the protein with the key amino acids Asp 197, Glu 233, Asp 197, Glu 233, Trp 59, Tyr 62, His 101, Leu 162, Arg 195, His 299 and Leu 165.

**Table 8:** Molecular docking value of flavonoids against Human α-amylase (PDB ID: 4W93).

Compound	*Affinity (kcal/mol)*	*H-b*ond	Residual Amino acid Interactions	
			Hydrophobic/Pi-Cation/Pi-Anion/ Pi-Alkyl interactions	Van-der Walls interactions
**1**	- 8.3	Gln 63	Tyr 62, Trp 58, His 299, Asp 300	Asn 298, Arg 195, Ile 235
**2**	-8.1	Asp 197, Glu 233	Trp 59, Tyr 62, Hoh 773, Hoh 789	Leu 165, His 101, Leu 162, Ala 198, Arg 195, Asp 300, His 299
**3**	-7.6	Gln 63	Trp 50 Tyr 62, Trp 58	Thr 163, Leu 165, His 299, Arg 195, Asn 298, Glu 233, Asp 300
**4**	-8.2	Arg 195, Asp 300	Trp 59, Trp 58, Tyr 62, His 299	Gln 63
**5**	-8.0	Glu 233	Trp58, Trp 59	His 305, Asp 300, Arg 195 Asp 197, His 101, Tyr 62, Leu 165, Gln 63
**6**	-7.8	Gln 63, Tyr 62 Glu 233	Trp 59, Tyr 62	Leu 165, Trp 58, His 299, Asp 197, Arg 195, Ala 198
**7**	-7.8	---	Tyr 62, Trp 59	Trp 58, His 299, Arg, 195, Asp 197, Ala 198, His 101, Gln 63
**8**	-8.1	Gln 63, Tyr 62	Trp 59, Tyr 62	Trp 58, His 299, Glu 233, Asp 197, Arg 195, Ala 198 Leu 165
**9**	-8.1	Gln 63, Tyr 62, Glu 233, Asp 197	Trp 59, Tyr 62	Leu 165, Ala 198, Arg 195, Trp 58, His 299
**10**	-7.7	Gln 63, Asp 197	Trp 59	Ala 198, Glu 233
**11**	-7.6	Gln 63, Asp 197	Trp 59, Tyr 62, Asp 197, Glu 233, Arg 195	Leu 165, His 101, Trp 58, His 299
**12**	-8.3	Gln 63	Trp 59, Tyr 62, Arg 195	Trp 58, His 299, Glu 233, Ala 198, Leu 165
**13**	-7.5	Gln 63	Tyr 62, Trp 59, Asp 300	Asp 197, Arg 195 His 299, Trp 58
**14**	-7.6	Gln 63, Arg 195, Glu 233	Trp 59, Tyr 62	Trp 58, His 299, Leu 165
**15**	-7.8	Arg 195 Asp 300	Trp 59 Tyr 62, His 299, Leu 162	Gln 63, Asp 197, Glu 233, Asn 298, ILE 235
**16**	-8.0	His 299, Gln 63, Asp 197	Trp 59, Asp 197, Tyr 62	Gln 63, His 101, Arg 195
**18**	-4.5	Asp 197, Glu 233	Asp 197, Glu 233	Trp 59, Tyr 62, Leu 165, His 101, Leu 162, Arg 195, His 299

The tested flavonoids showed a binding energy ranging from -7.5 to -8.3 kcal/mol. All the investigated compounds showed strong binding score compared to the clinical drug metformin (**18**) with -4.5 kcal/mol. The top seven flavonoids with low binding energies were apigenin (**1**), baicalein (**2**), chrysin (**4**), ellagic acid (**5**), kaempferol (**8**), quercetin (**9**), and luteolin (**12**). A previous work reported that compound quercetin (**9**) has low binding energy (-8.0 kcal/mol) against α-glucosidase [74], inferring the inhibitory potential of the compound towards α-amylase and α-glucosidase. Overall, the *in-silico* docking analysis indicated that apigenin (**1)** that interacts with the α-amylase residues of Tyr 62, Trp 58, His 299, Asp 300, Asn 298, Arg 195 and Ile 235 with binding energy -8.3 kcal/mol, chrysin (**4)** that interacts with the α-amylase residues of Trp 58, Trp 59, Tyr 62, His 299 and Gln 63 with binding energy -8.1 kcal/mol, kaempferol (**8)** that interacts with the α-amylase residues of Trp 58, Trp 59, Tyr 62, His 299, Glu 233, Asp 197, Arg 195, Ala 198, and Leu 165 with binding energy -8.1 kcal/mol, quercetin (**9**) (-8.1 kcal/mol), and luteolin (**12**) that interacts with the α-amylase residues of Trp 59, Tyr 62, Arg 195, Trp 58, His 299, Glu 233, Ala 198, and Leu 165–8.3 kcal/mol are better anti-diabetic agents compared to other compounds reported in this study.

The lower binding energies predicted for compounds apigenin (**1**), chrysin (**4**), kaempferol (**8**), quercetin (**9**), luteolin (**12**) relative to the control metformin (**18)** also indicated that they fit well in the binding pocket of the human α-amylase forming a stable inhibitor-protein complex.

## Conclusions

In this study we presented the pharmacokinetic, drug-likeness, and toxicity profile of sixteen flavonoid derivatives and two control drugs (metformin (**18**) and acarbose (**17**)). The results show appropriate logP, LogS, and high HIA values for chrysin (**4**), wogonin (**15**), baicalein (**2**), genistein (**10**) and apigenin (**1**), suggesting better absorption and less toxicity. This has an implication that the flavonoids can be used as bidentate metal complexing agents, of which chrysin (**4**) was found to be more ideal for metal complexation with high absorption and lipophilicity with 84.6% absorption compared to metformin (**18**) (78.3%). The tendency of chrysin (**4**) to cross BBB and act on CNS was also predicted using SwissADME and ADMETlab predictions. Baicalein (**2**), butein (**3**), ellagic acid (**5**), eriodyctiol (**6**), fisetin (**7**) and quercetin (**9**) were predicted to show carcinogenicity. Most of the molecules are predicted as CYP3A probes, except eriodyctiol (**6**) which interacts with p-gp. Toxicological endpoints prediction analysis indicated median lethal dose (LD_50_) values ranging from 159–3919 mg/Kg, of which baicalein (**2**) and quercetin (**9**) were found to be mutagenic, whereas butein (**3**) and morin (**15**) were found to be immunotoxin among the studied flavonoid derivatives. Based on the findings from the predicted drug-likeness, toxicity, pharmacokinetic properties together with quantum chemical descriptors and molecular docking, chrysin (**4**) is found to be a molecule of potential chelating agent to synthesize coordination compounds for further *in vitro* and *in vivo* studies. Moreover, the findings from the DFT calculated quantum chemical reactivity descriptors indicated that chrysin (**4**) is most likely to interact with metal ions to form stable coordination compounds than the other flavonoids. Overall, the results obtained in this study showed better pharmacokinetics and drug likeness profiles for chrysin (**4**), wogonin (**15**), genistein (**10**), baicalein (**2**) and apigenin (**1**), and showed toxicity alerts to the toxicological endpoints (above class three toxicity). However, further detailed experimental study is required to confirm the findings reported in this work.

## Supporting information

S1 File (DOCX)

S2 File The 2D and 3D representations of the binding modes as well as the xyz coordinates of the optimized structures are presented in the file. (XYZ)
