# Supplementary material for: Pharmacokinetics and drug-likeness of antidiabetic flavonoids: Molecular docking and DFT study
Source: PLoS One. 2021 Dec 10;16(12):e0260853. doi: 10.1371/journal.pone.0260853 (PMC8664201; doi:10.1371/journal.pone.0260853)
Supplement: S1 File — (DOCX) [file pone.0260853.s001.docx]

**Pharmacokinetics and Drug-Likeness of Antidiabetic Flavonoids: Molecular Docking and DFT Study**

Mamaru Bitew^1^*, Tegene Desalegn^1^, Taye B. Demissie^2^, Anteneh Belayneh^3^, Milkyas Endale^1^, Rajalakshmanan Eswaramoorthy^1^*

**^1^** Program of Applied Chemistry, School of Applied Natural Science, Adama Science and Technology University, Adama, Oromia Regional State, Ethiopia**.**

**^2^** Department of Chemistry, University of Botswana, Notwane Rd, P/bag UB 00704 Gaborone, Botswana**.**

^3^ Department of Pharmacy, College of Health Science, Debre Markos University, Debre Markos, Amhara Regional state, Ethiopia**.**

*Correspondence E-mail: [mamaru2005@gmail.com](mailto:mamaru2005@gmail.com), [rajalakshmanan.e@gmail.com](mailto:rajalakshmanan.e@gmail.com)

**Table of Contents**

[**Fig S 1.** 2D and 3D representations of the binding modes of compounds Biacalien and Butien to Human α-amylase. 2](#_Toc77608821)

[**Fig S 2**.2D and 3D representations of the binding modes of Ellagic acid and Eriodyctiol. 4](#_Toc77608822)

[**Fig S 3**. 2D and 3D representations of the binding modes of Fisetin and Kaempferol. 5](#_Toc77608823)

[**Fig S 4.** 2D and 3D representations of the binding modes of Quercetin and Genistein. 6](#_Toc77608824)

[**Fig S 5.** 2D and 3D representations of the binding modes of Isorhamnetin and Luteolin. 7](#_Toc77608825)

[**Fig S 6.** 2D and 3D representations of the binding modes of Morin and Naringenin 8](#_Toc77608826)

[**Fig S 7.** 2D and 3D representations of the binding modes of Wogonin and Myricetin 9](#_Toc77608827)


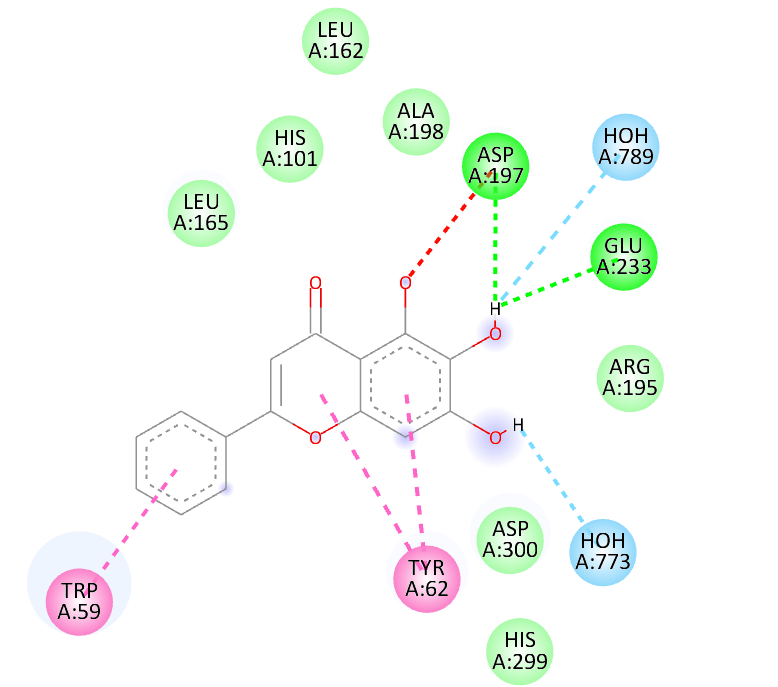

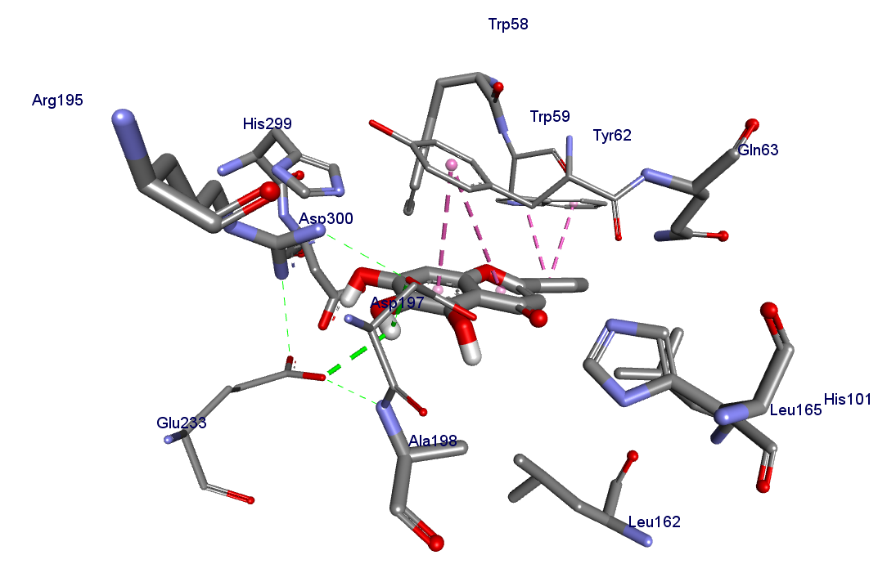


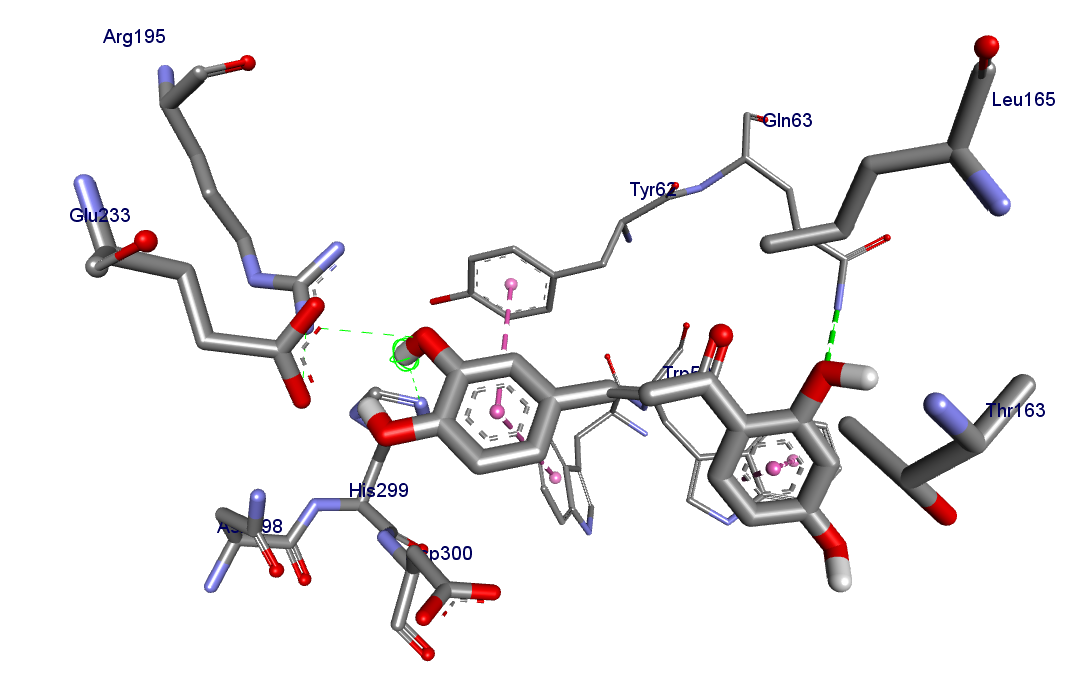


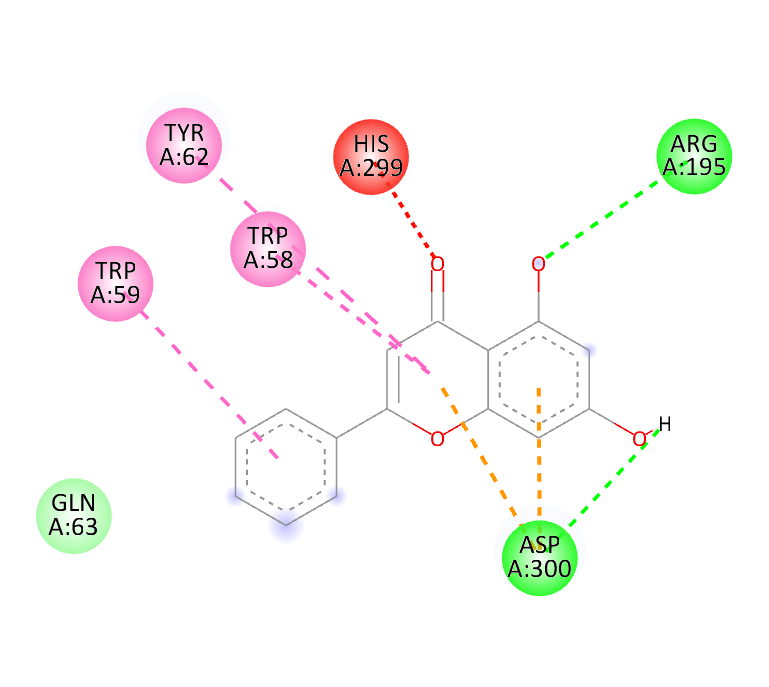


**Fig S 1.** 2D and 3D representations of the binding modes of compounds Biacalien and Butien to Human α-amylase.


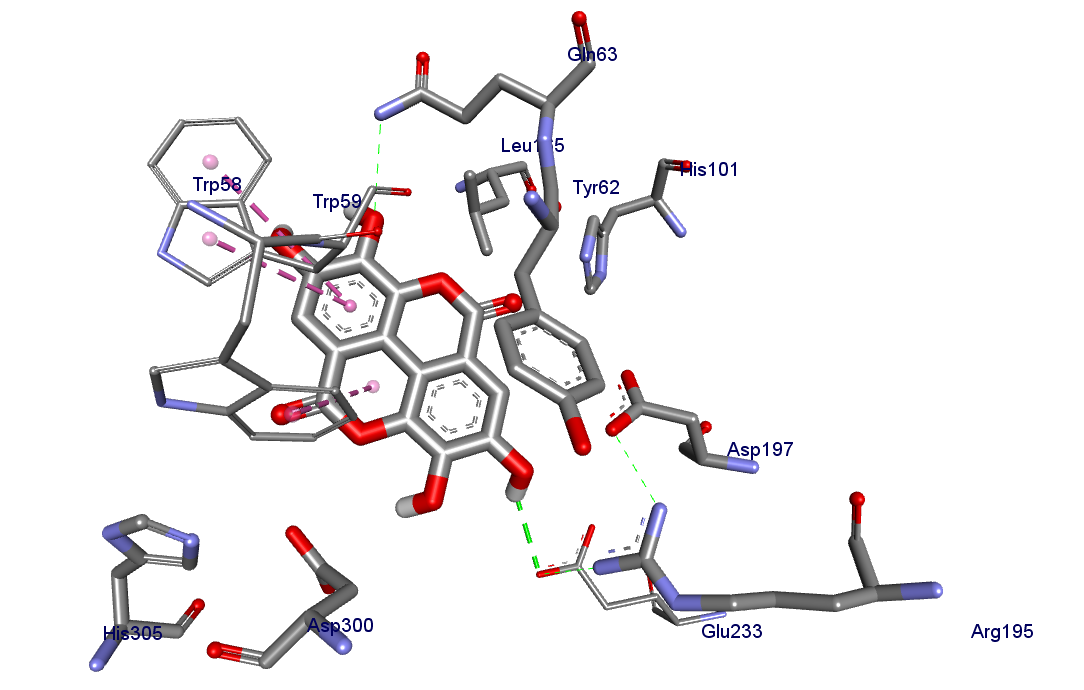

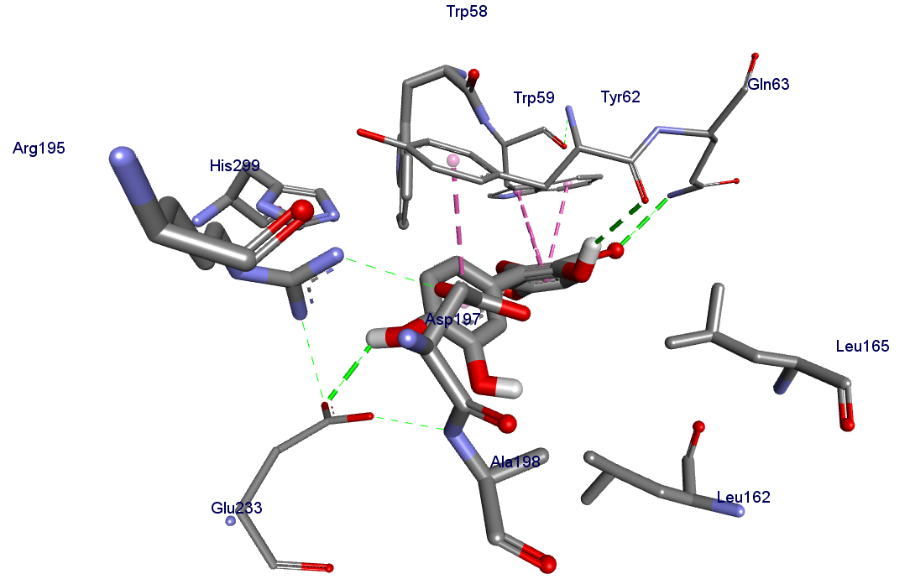

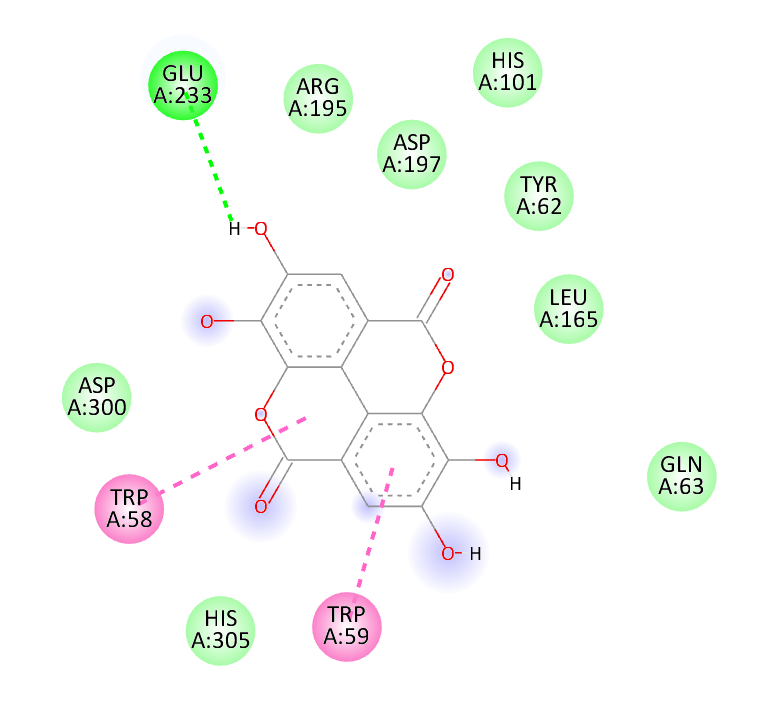


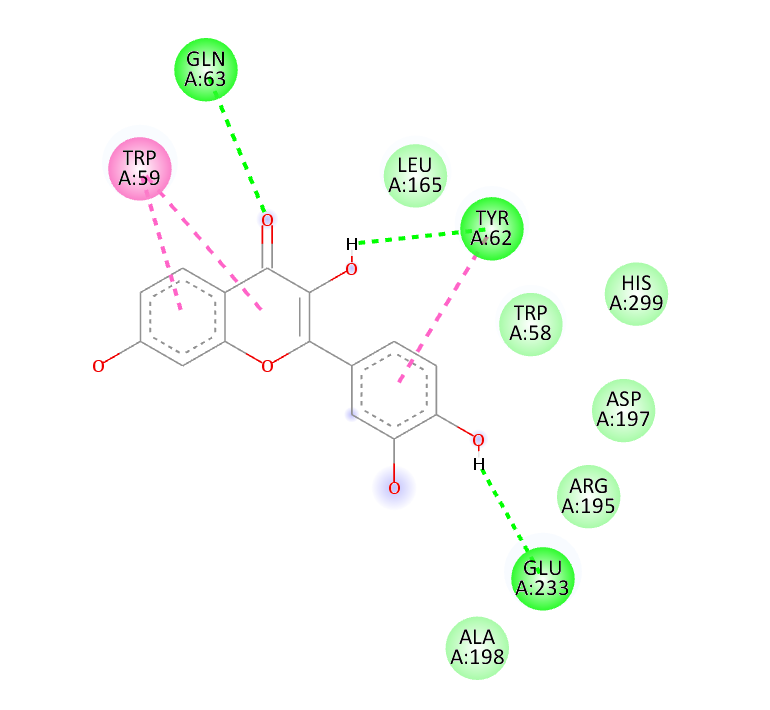


**Fig S 2**.2D and 3D representations of the binding modes of Ellagic acid and Eriodyctiol.


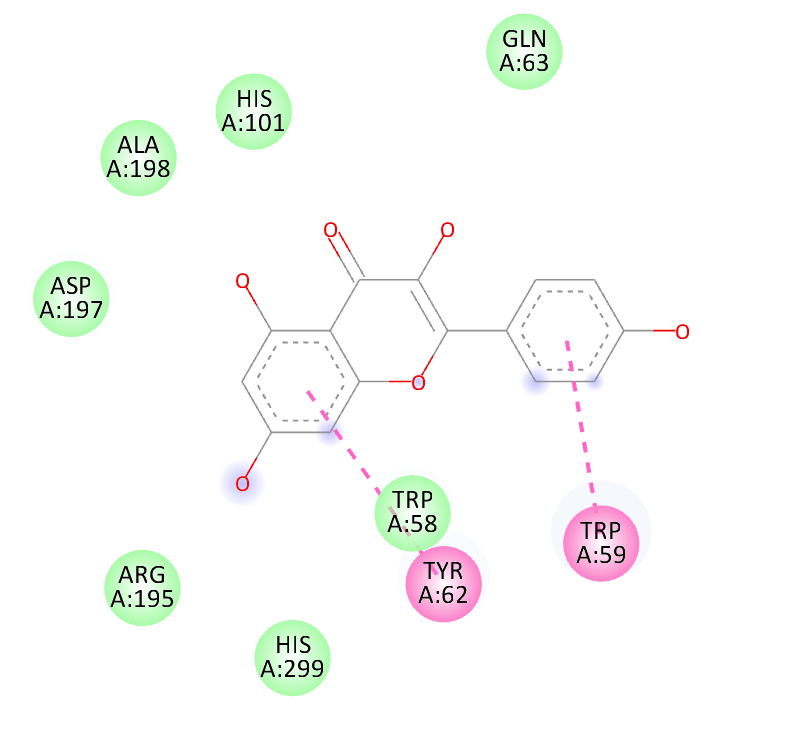

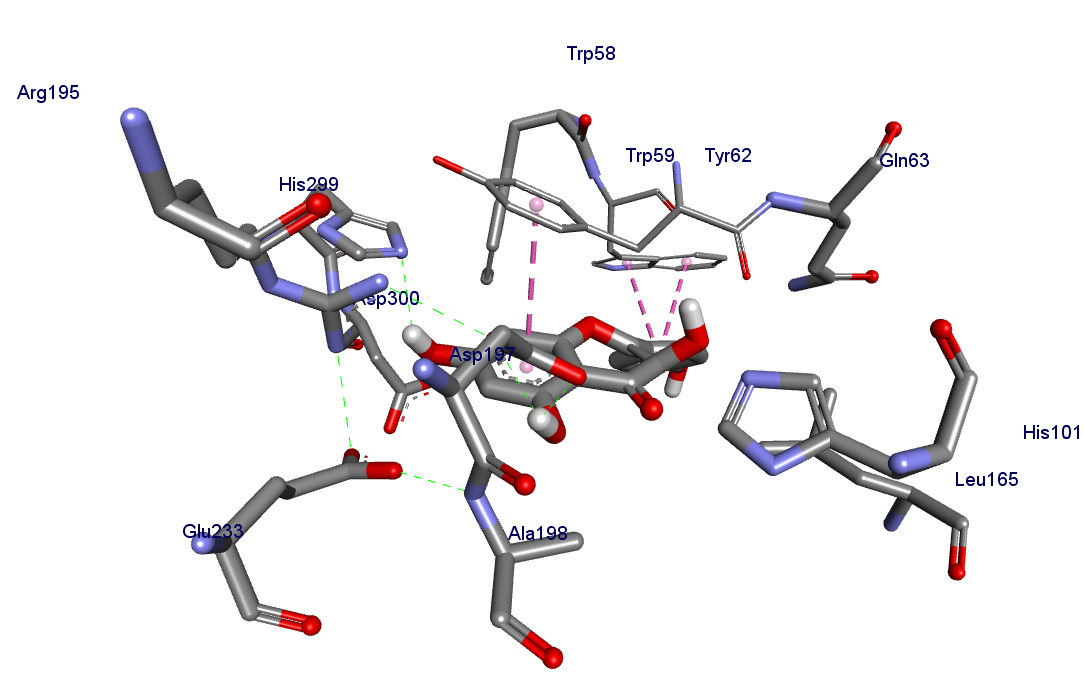

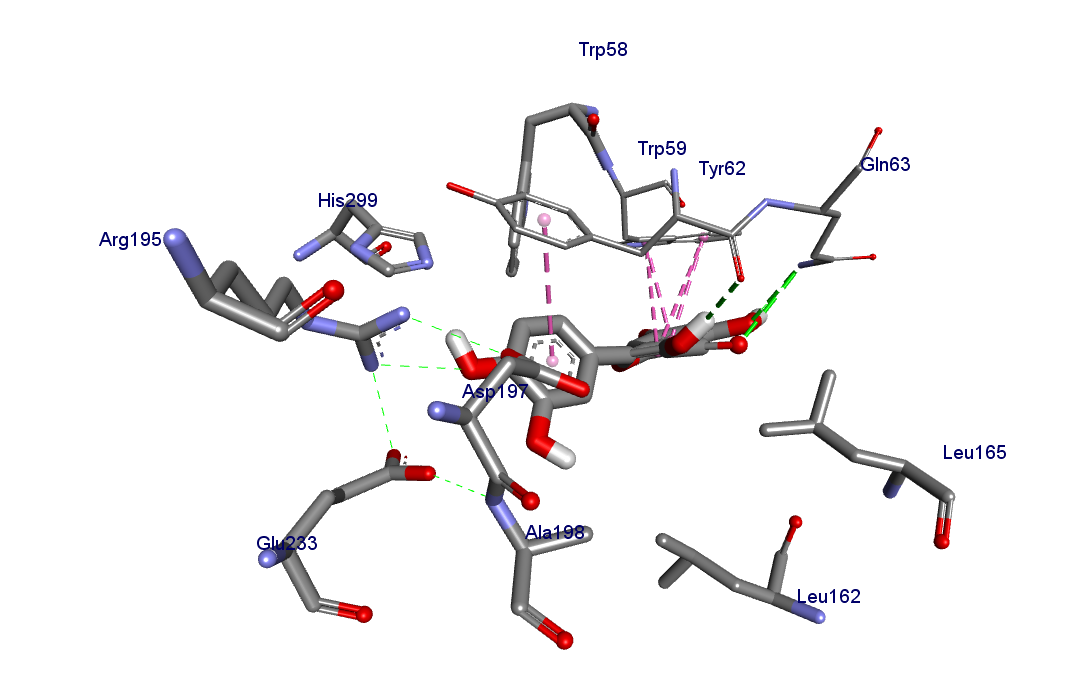

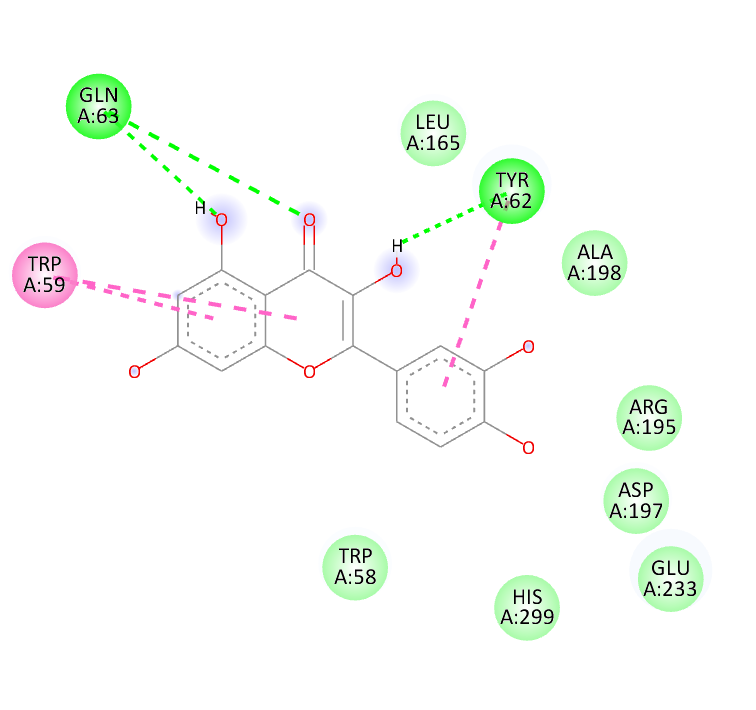


**Fig S 3**. 2D and 3D representations of the binding modes of Fisetin and Kaempferol.


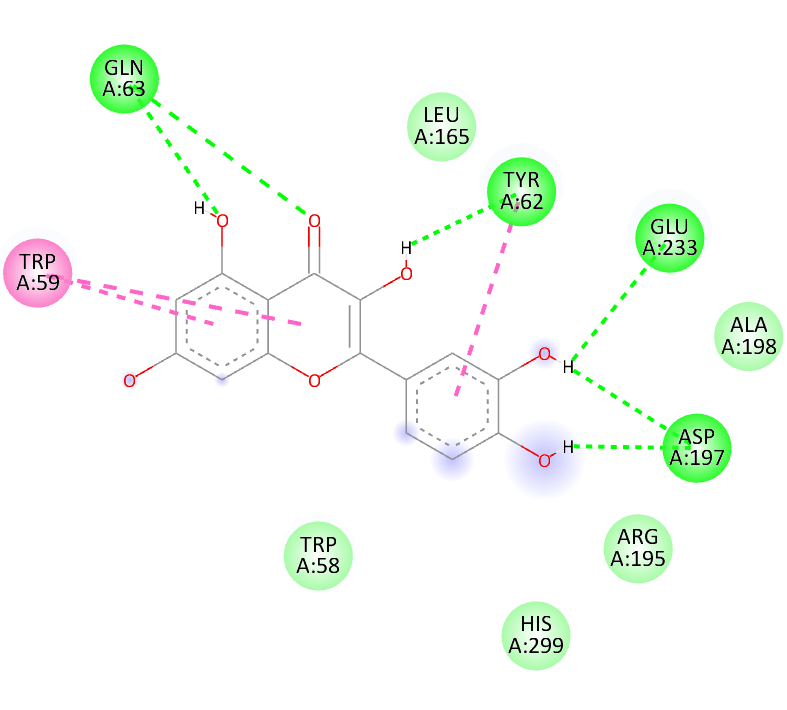

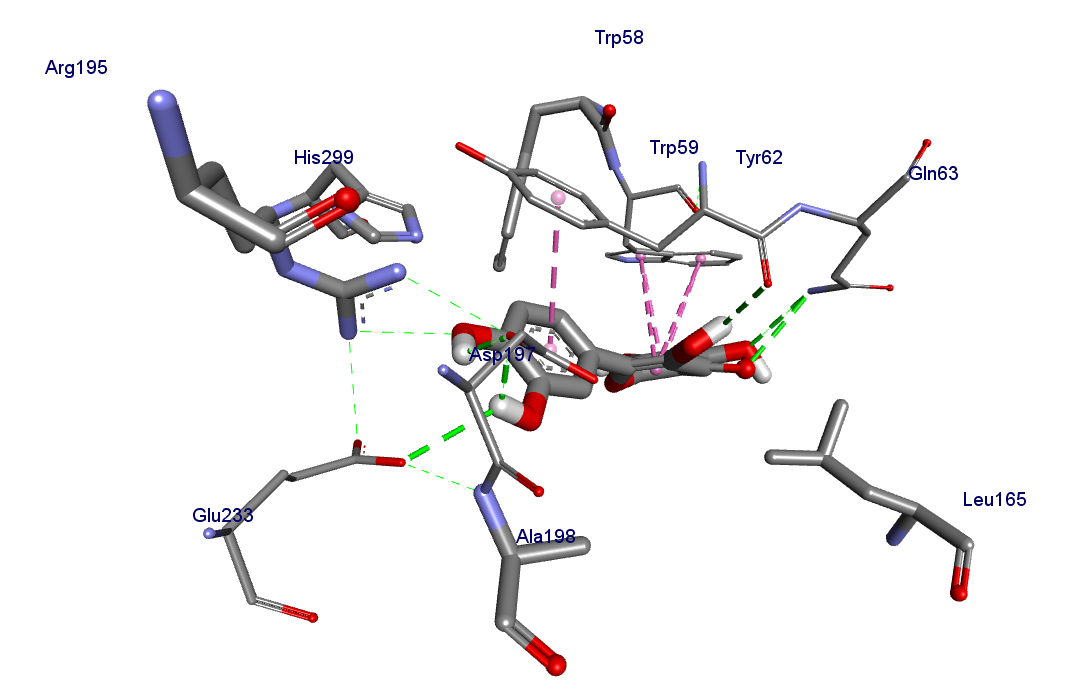


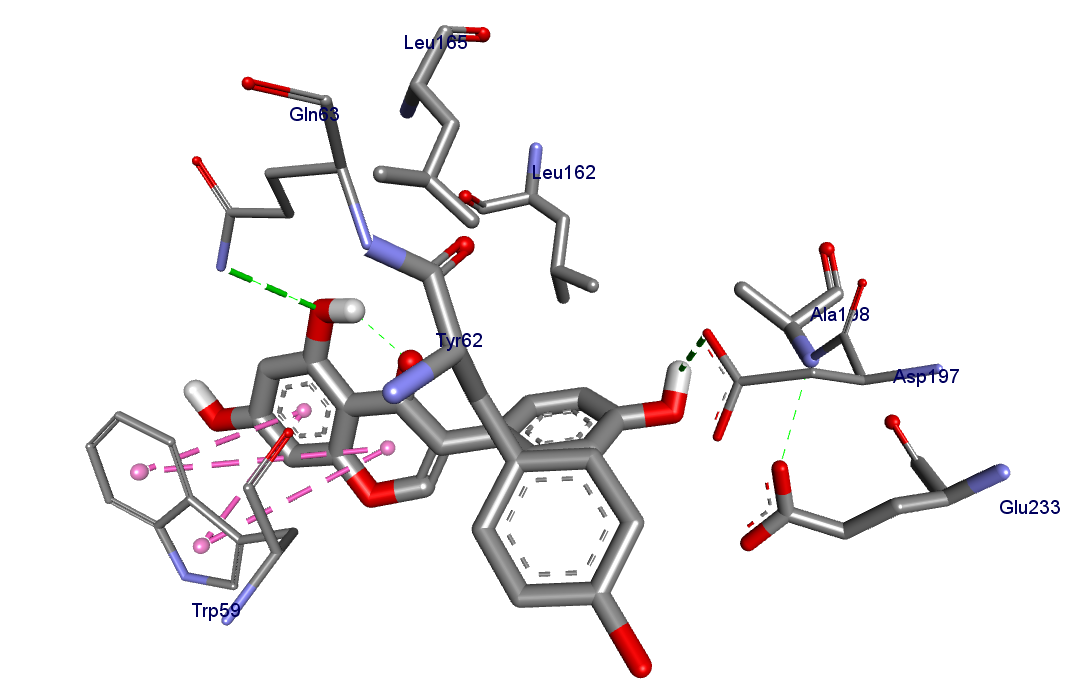

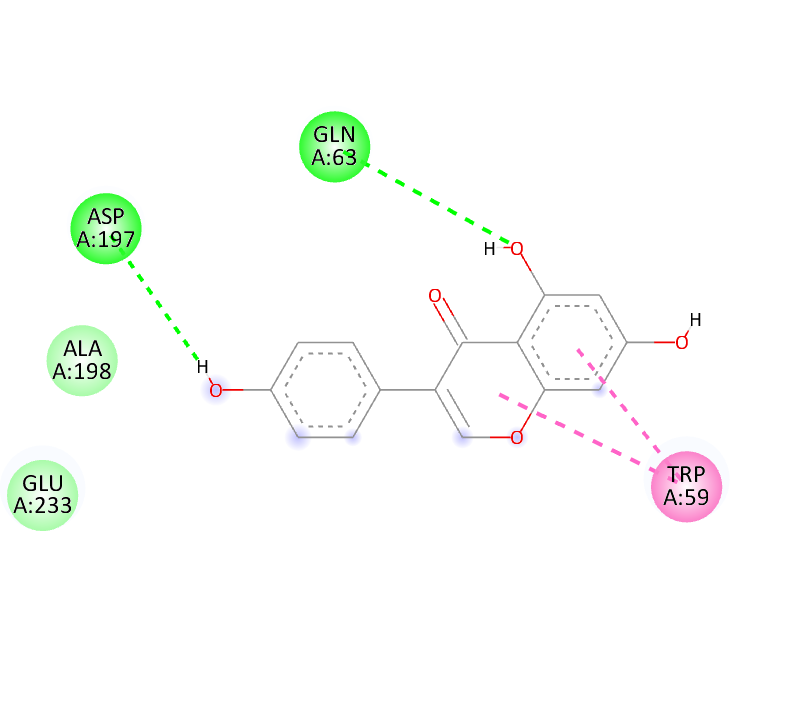


**Fig S 4.** 2D and 3D representations of the binding modes of Quercetin and Genistein.


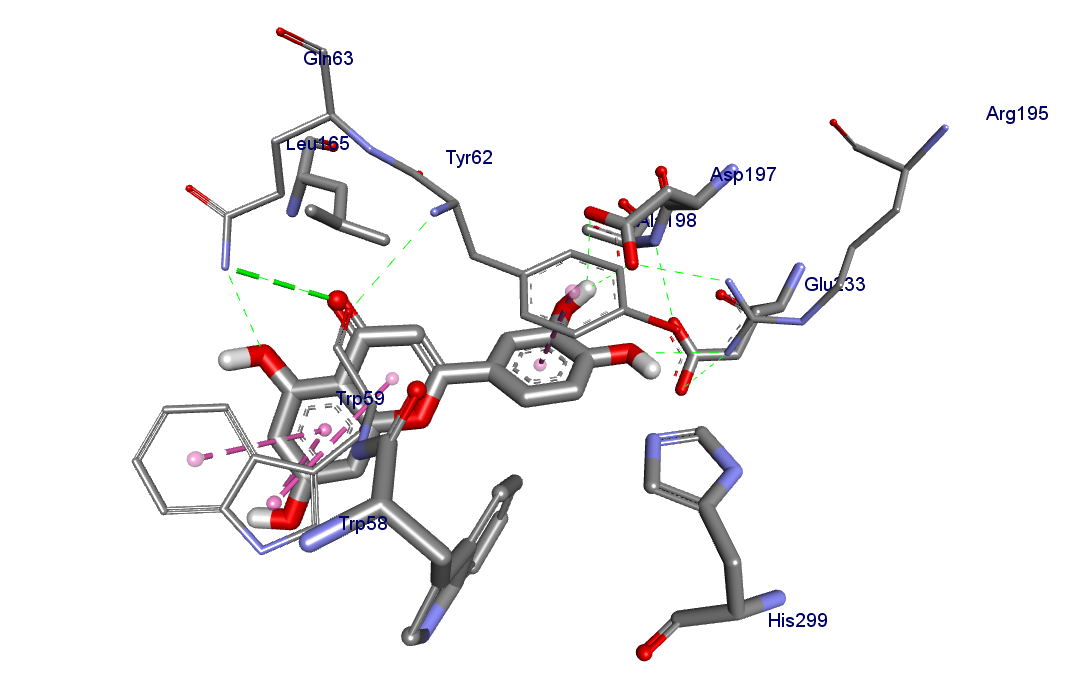

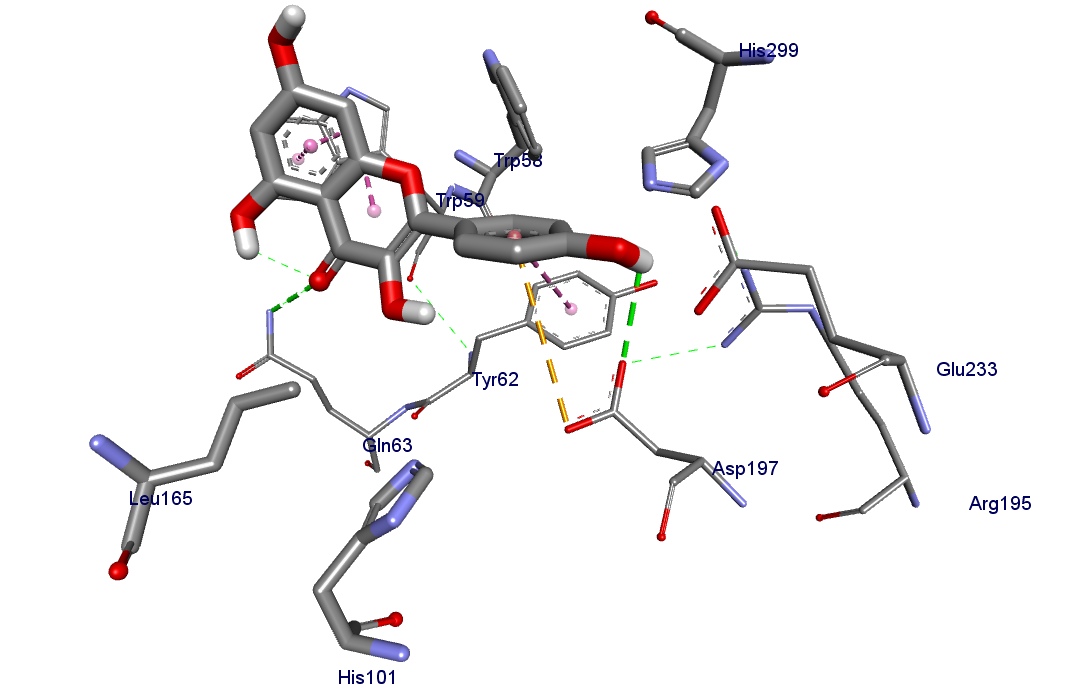

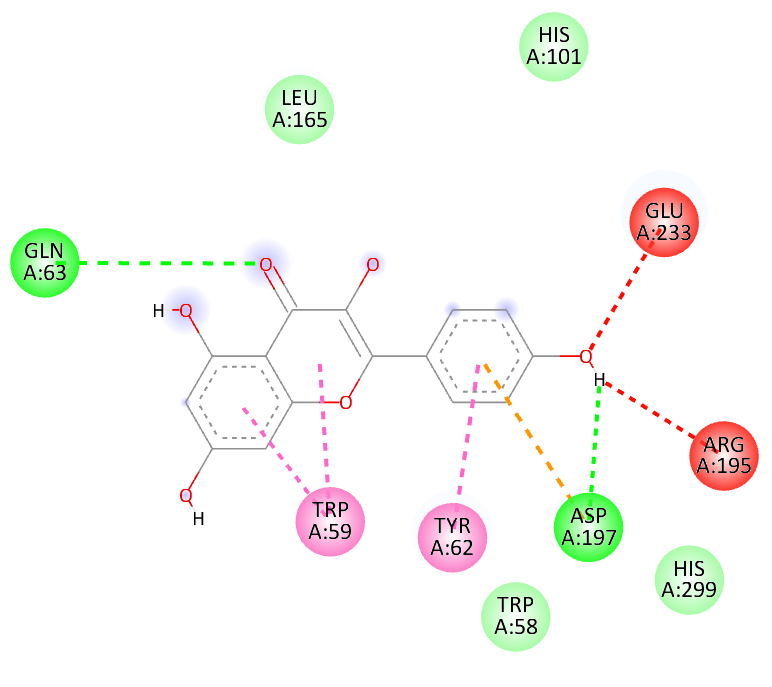

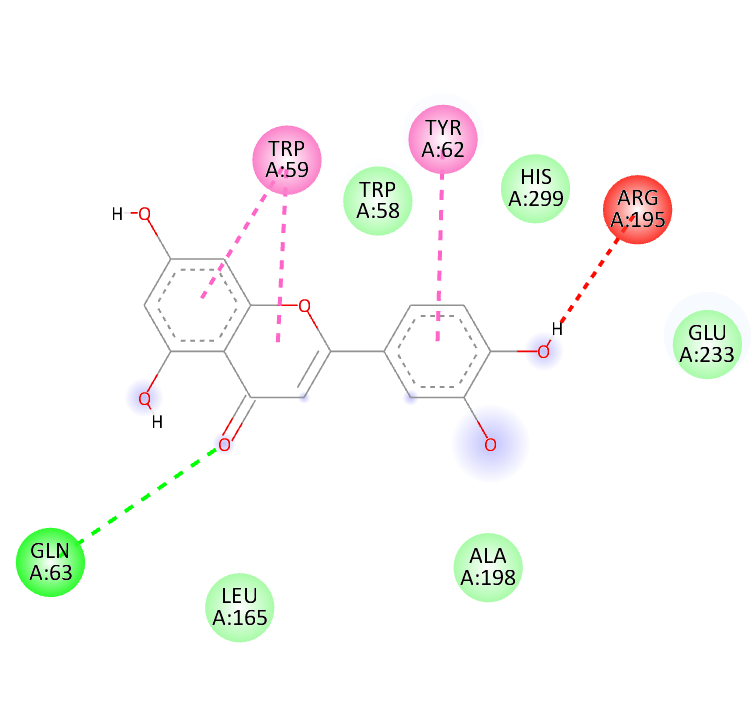


**Fig S 5.** 2D and 3D representations of the binding modes of Isorhamnetin and Luteolin.


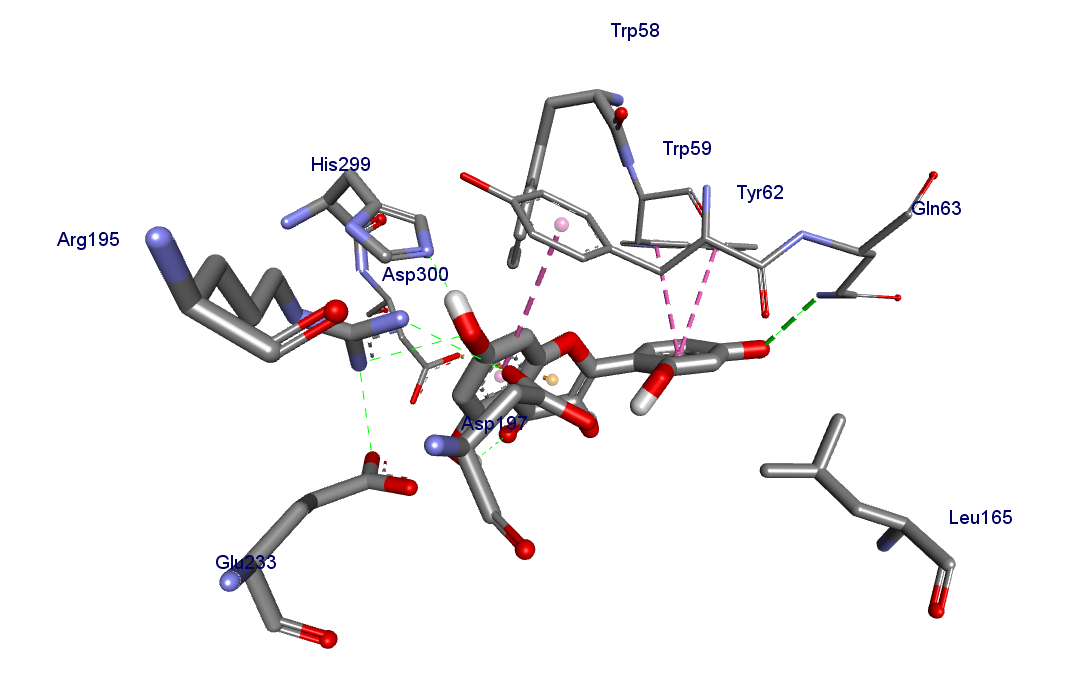

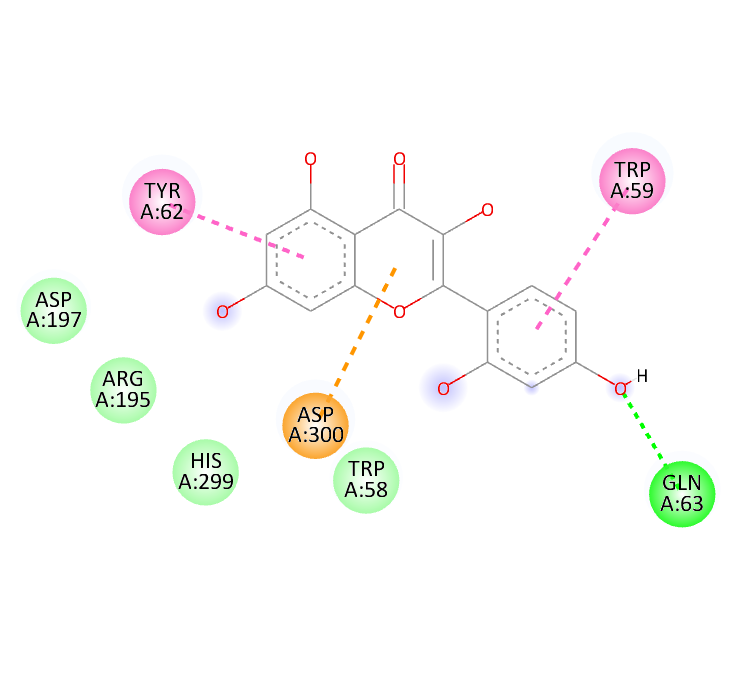


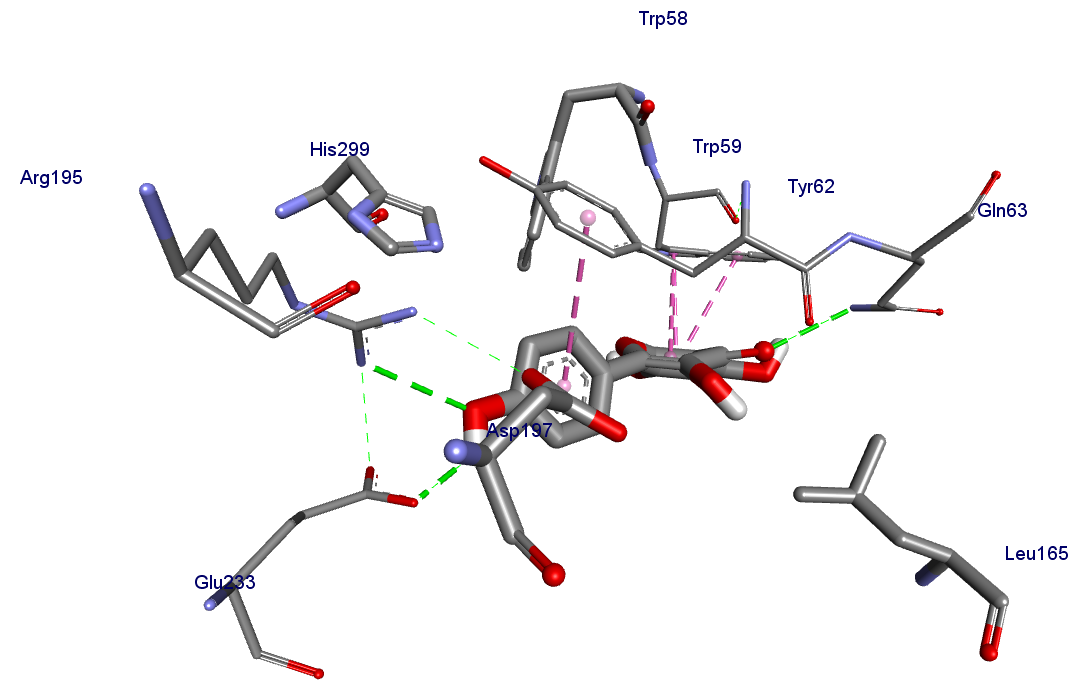

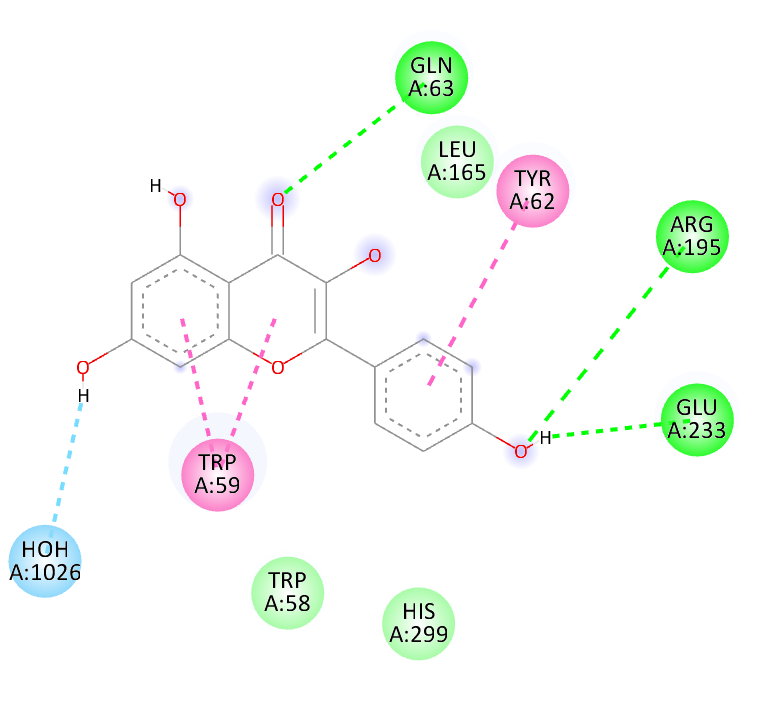


**Fig S 6.** 2D and 3D representations of the binding modes of Morin and Naringenin


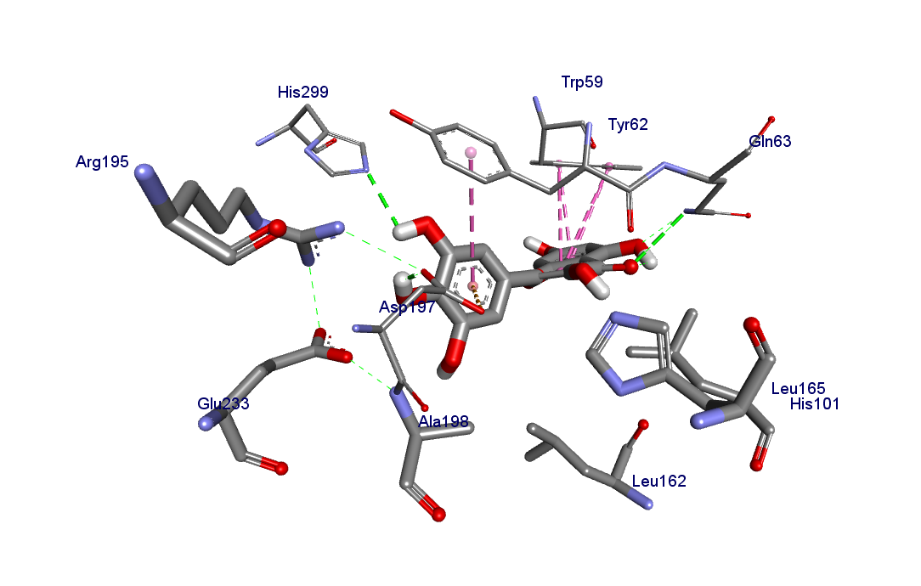

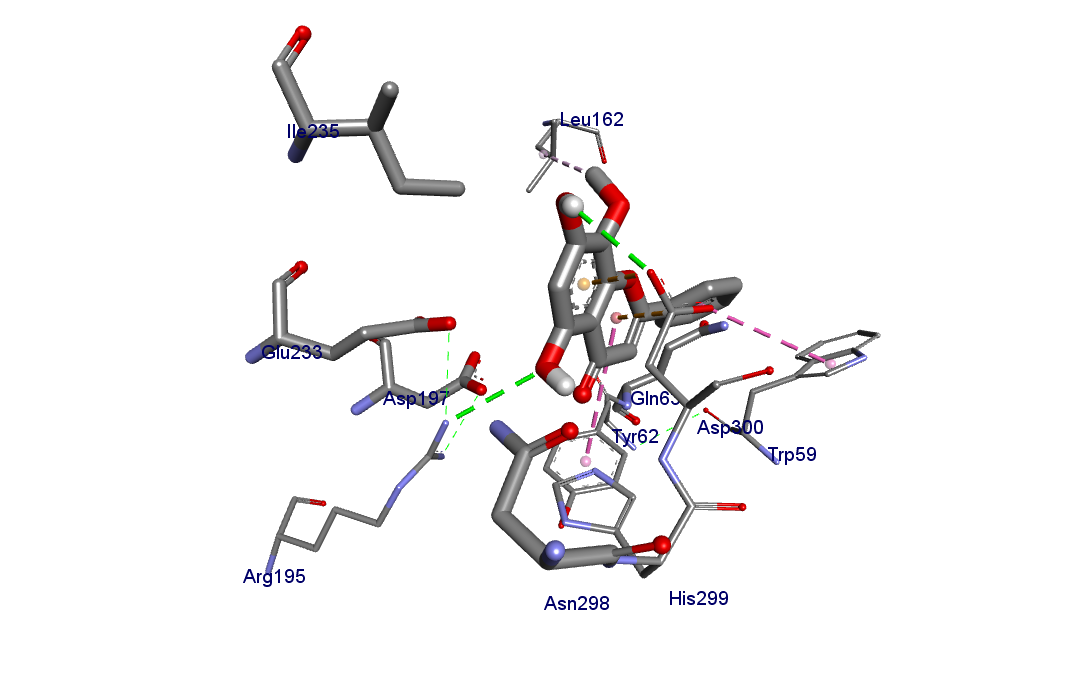

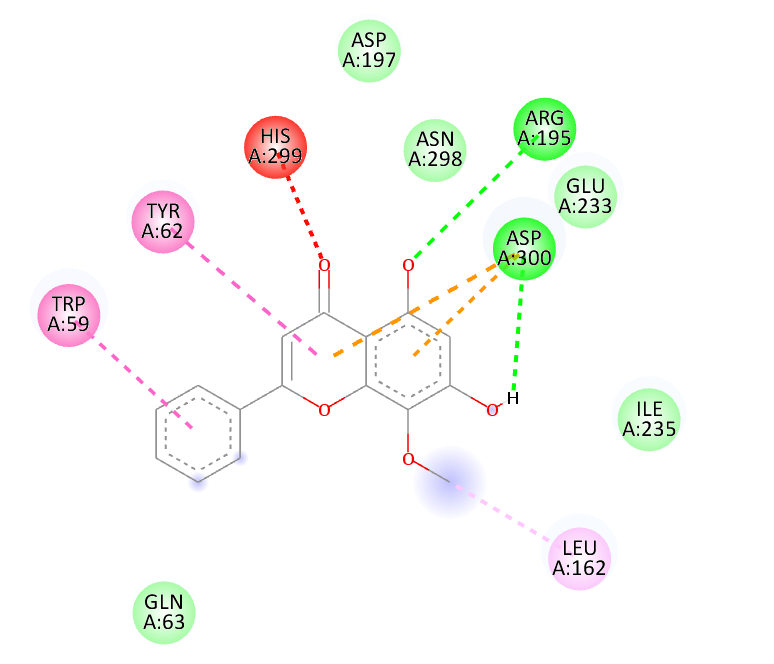

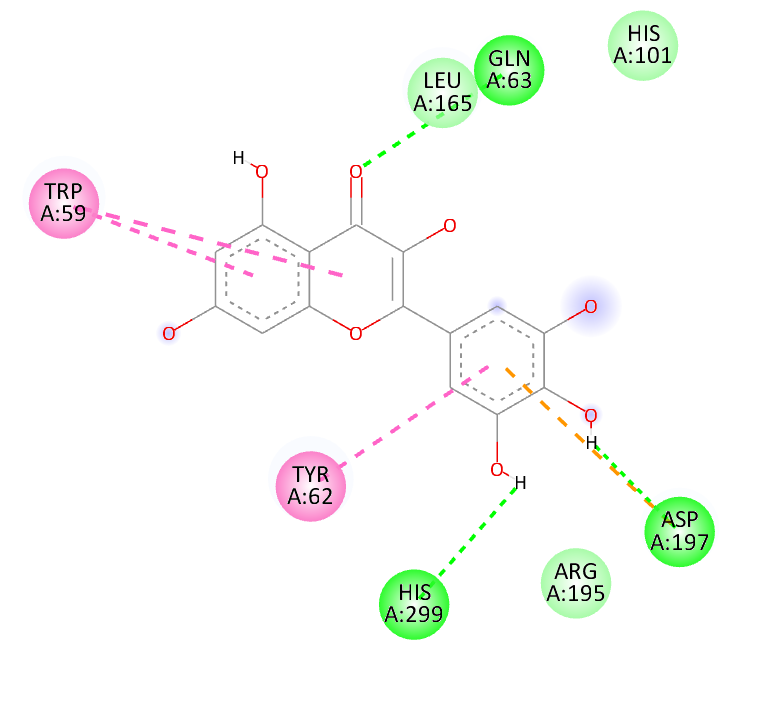


**Fig S 7.** 2D and 3D representations of the binding modes of Wogonin and Myricetin
